# Identifying the copper coordination environment between interacting neurodegenerative proteins: A new approach using pulsed EPR with ^14^N/^15^N isotopic labeling

**DOI:** 10.1016/j.jbc.2025.108311

**Published:** 2025-02-13

**Authors:** Amanda Smart, Kevin Singewald, Zikri Hasanbasri, R. David Britt, Glenn L. Millhauser

**Affiliations:** 1 Department of Chemistry and Biochemistry, University of California Santa Cruz, Santa Cruz, California, United States; 2 Department of Chemistry, University of California Davis, Davis, California, United States

**Keywords:** EPR, Alzheimer's disease, amyloid-beta, prion protein, copper, ternary complex

## Abstract

The trafficking and aggregation of neurodegenerative proteins often involve the interaction between intrinsically disordered domains, stabilized by the inclusion of physiological metal ions such as copper or zinc. Characterizing the metal ion coordination environment is critical for assessing the stability and organization of these relevant protein-protein interactions but is challenging given the lack of regular molecular order or global structure. The cellular prion protein (PrP^C^) binds both monomers and aggregates of Alzheimer's amyloid-beta (Aβ), promoting A**β** internalization and aberrant signaling, respectively. Both proteins bind Cu^2+^ with high affinity, opening the potential for copper to form an intermolecular bridge. We describe here a novel approach utilizing multiple EPR experiments to investigate the simultaneous Cu^2+^ coordination of PrP^C^ and A**β** in a 1:1:1 mixture. Uniformly ^15^N-labeled PrP^C^ is used in conjunction with natural abundance ^14^N A**β**, the combination of which leads to distinct energy manifolds for paramagnetic Cu^2+^ and is resolved by the pulsed EPR experiments ESEEM and HYSCORE. We develop acquisition parameters to simultaneously optimize ^14^N (I = 1) and ^15^N (I = ½) pulsed EPR signals and we also advance the theory of ESEEM and HYSCORE to quantitatively describe multiple ^15^N imidazole coordination. This unique approach provides compelling evidence of a copper-stabilized ternary complex, with equatorial Cu^2+^ coordination formed by one histidine imidazole from A**β** and three from PrP. Moreover, the methodologies developed here provide a framework for assessing the copper environment in other interacting neurodegenerative proteins.

Neurodegenerative diseases are initiated by protein-protein interactions, typically in the form of extracellular aggregates. In turn, these aggregates trigger downstream events ultimately compromising neuronal function. Alzheimer's disease (AD) begins with intermolecular association of Aβ, a 40- to 42-amino acid peptide derived from proteolytic cleavage of the amyloid precursor protein (APP) (1). Interestingly, the cellular prion protein (PrP^C^), which itself causes a separate set of neurodegenerative diseases, is recognized as a high-affinity receptor for both monomeric and oligomeric Aβ, the latter of which is designated Aβo (2, 3, 4, 5, 6). Monomeric Aβ binding to PrP^C^ results in the translocation of Aβ through endocytosis, possibly leading to its intracellular accumulation (4, 7). Aβo binding to PrP^C^ is proposed to trigger aberrant transmembrane signaling through the metabotropic glutamate receptor 5 (mGluR5) ultimately causing intracellular tau aggregation and the formation of neurofibrillary tangles (6, 8, 9). Metal ion binding is likely to play a significant role in all these processes. Senile plaques formed primarily by Aβ aggregates are rich in both copper and zinc (10, 11, 12, 13, 14). The occlusion of these metal ions is proposed to both stabilize aggregate structure and contribute to deleterious chemical reactions through the formation of reactive oxygen species (15, 16, 17, 18, 19). In their monomeric forms, both Aβ and PrP^C^ bind Cu^2+^ with high affinity (10, 18, 20). As such, assessment of the interaction between these two proteins must consider their respective metalloprotein characteristics.

Murine PrP^C^ is composed of 208 amino acids (residues 23–230), post-translationally modified with a C-terminal glycophosphatidylinositol anchor and two Asn-linked glycans at residues 180 and 196. The N-terminal segment (residues 23–125, after removal of the signal peptide) is an intrinsically disordered peptide (IDP), whereas the C-terminal domain is primarily helical. Within the N-terminal IDP is the octapeptide repeat (OR) domain, residues 59 to 90, composed of the sequence (PHGGXWGQ)_4_ in mouse PrP^C^ (where X is Gly in repeats one and four, Ser in repeats two and three), which binds up to four equivalents of Cu^2+^ (21, 22). It is now established that Cu^2+^ forms a coordination bridge between the OR domain and the C-terminal domain; the stability of this bridge is further enhanced by glycosylation (23, 24, 25). Without this critical N-terminal—C-terminal interaction, the N-terminal domain is untethered and thus drives highly cytotoxic membrane leakage (26).

We demonstrated in previous work that PrP^C^ translocates monomeric Aβ across the plasma membrane by endocytosis (4). Assays were performed with both Aβ(1–40) and Aβ(1–30), the latter of which lacks the peptide's hydrophobic C-terminal segment and thus remains soluble. By switching Aβ stereochemistry, we showed that its translocation requires a direct protein-protein interaction. Aβ(1–30) is flexible, possesses three His residues within its first 14 amino acids, and, similar to PrP^C^, avidly binds Cu^2+^ (4, 19).

Given the demonstrated interaction between PrP^C^ and Aβ and the fact that both are rich with His residues, we propose that Cu^2+^ promotes intermolecular coordination linkage between the proteins. However, assessing metal ion coordination involving IDP segments in protein complexes is refractory to traditional methods that depend on well-defined protein structures or long-range order. The EPR methods of electron spin echo envelope modulation (ESEEM) (27) and hyperfine sublevel correlation (HYSCORE) (28) are ideal for assessing His coordination (29, 30, 31), however, they cannot distinguish which protein is presenting a specific His residue. Here we develop a new strategy for determining how Cu^2+^ stabilizes intermolecular interactions using Aβ-PrP^C^ as a model system. Our approach combines the pulsed EPR techniques of ESEEM and HYSCORE along with ^15^N isotopic labeling of PrP^C^, thus enabling the assignment of spectroscopic signals to residues of the specific interacting partner. We develop data acquisition parameters to produce high-quality spectra simultaneously from ^14^N and ^15^N pulsed EPR signals. We also expand the theory of ^15^N pulsed EPR to account for multiple His residue coordination. Our results not only provide a compelling model for how Cu^2+^ stabilizes the interaction between Aβ and PrP^C^, but they also yield a systematic EPR methodology for assessing Cu^2+^ coordination in more general protein complexes.

## Results and discussion

### Copper coordination to PrP^C^ and A**β**

**Figure 1:**
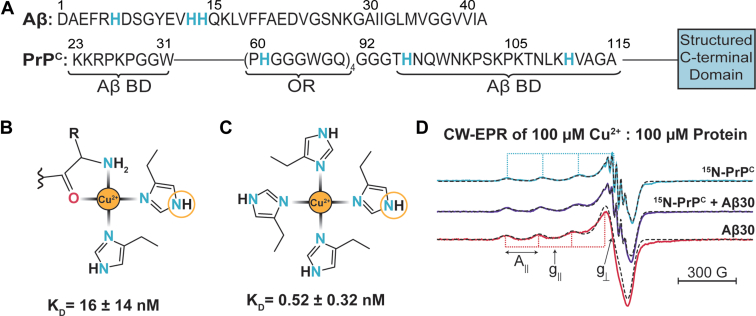
**Continuous-wave EPR spectra of Cu**^**2+**^**coordinating to Aβ30 and PrP**^**C**^**.***A*, the linear sequence for Aβ (*top*) and PrP^C^ (*bottom*) are shown. Histidine residues that participate in Cu^2+^ binding are *blue* and *bolded*. Both Aβ and the N-terminus of PrP^C^ (residues 23–125) are intrinsically disordered. The C-terminus of PrP^C^ (residues 126–230) is structured and represented as the *blue box*. The two putative Aβ binding domains (Aβ BD) and Cu^2+^ binding domain known as the octapeptide repeat (OR) domain are labeled in PrP^C^. *B*, the consensus Aβ-Cu^2+^ coordination environment at physiological pH consists of two histidine residues, a backbone carbonyl, and the N-terminal amine. *C*, the PrP^C^- Cu^2+^ coordination at physiological pH and at equimolar ratio consist of four histidine residues, all from the OR domain. The corresponding affinity (reported as K_D_ values) for the shown coordination state is listed below (*B* and *C*). The circled nitrogen on the histidine, known as the remote nitrogen or distally coupled nitrogen, is monitored by ESEEM and HYSCORE EPR. *D*, the stacked CW of ^15^N-PrP^C^ (*blue*), ^15^N-PrP^C^:Aβ30 (*purple*) and Aβ30 (*red*) are all measured with one equivalent copper at pH 7.4. The sharp, high field features are due to superhyperfine splitting from directly coordinated ^15^N. The dashed spectra overlayed are the respective simulated spectrum. A_∥_ is shown as the separation between the low field peaks, indicated by the *blue* (PrP^C^) and red (Aβ30) *dotted lines*. The fields related to g_∥_ and g_⊥_ are labeled.

The linear sequences of Aβ and PrP^C^ are shown in Figure 1*A*. Aβ is a 40- to 42-amino acid-long IDP that quickly aggregates, making it difficult to study in biophysical assays. To overcome this tendency to aggregate, a truncated form of Aβ, consisting of the first 30 residues, is used. This truncated Aβ, referred to as Aβ30, is the longest non-aggregating fragment found that retains the ability to bind to both Cu^2+^ and PrP^C^ (4). PrP^C^ has an intrinsically disordered N-terminal domain (residues 23–125) and a structured C-terminal domain (residues 126–230). The flexibility of the N-terminal domain allows for the protein to interact with multiple binding partners, as well as fold back onto the structured domain in the presence of Cu^2+^ (24, 25, 26, 32, 33, 34, 35, 36, 37, 38). Within the N-terminal domain of PrP^C^, there are two proposed Aβ binding domains at residues 23 to 31 and 95 to 115 (2, 8, 39, 40) as well as the OR domain, a 4× repeated sequence each containing a metal binding histidine (20, 21). The OR domain can bind up to four Cu^2+^ ions; however, PrP^C^ can bind up to six equivalents of Cu^2+^ simultaneously with additional N-terminal sites at H95 and H110 (38, 41). In Figure 1*B*, we show the coordination of Aβ to Cu^2+^ at physiological pH. This coordination environment consists of the N-terminal amine, a backbone carbonyl, and histidine at residue six and residue 13 or 14 (10, 42). Although PrP^C^ can bind up to six copper ions at once, conditions used in this work, *i.e.*, 1 molar equivalence of Cu^2+^ to PrP^C^ at physiological pH results in one Cu^2+^ per PrP^C^ (21). The coordination of PrP^C^ to Cu^2+^ under these conditions consists of four histidine residues, one from each His in the OR domain, shown in Figure 1*C*. It should be noted that the binding affinity of Cu^2+^ for PrP^C^ is about 30-fold greater than that of Aβ30. Specifically, as calculated from the glycine competition assay in Fig. S2, the binding affinities of Cu^2+^ to Aβ30 and PrP^C^ under the buffer conditions in this study are 16.0 ± 14 nM and 0.519 ± 0.32 nM, respectively. These values fall well within the range of previous work (19, 20).

For the following continuous wave (CW) X-band EPR experiments, PrP^C^ was isotopically labeled with ^15^N, whereas Aβ30 was expressed without isotopic labeling. The use of ^15^N or natural abundance does not influence the copper A_∥_ and g_∥_ values obtained from CW-EPR, as shown in Fig. S3. In Figure 1*D*, the top trace is ^15^N-PrP^C^ with A_∥_ = 174 G and g_∥_ = 2.252. The bottom trace is Aβ30 with A_∥_ = 163 G and g_∥_ = 2.269. Both spectra report similar A_∥_ and g_∥_ values to previous work (10, 21, 43). Notably, the ^15^N-PrP^C^ spectrum represents a highly homogeneous Cu^2+^ coordination environment that reveals superhyperfine splitting in the perpendicular spectral region. By comparison, the Aβ30 spectra shows no superhyperfine splitting. Finally, the middle trace is the combination sample containing ^15^N-PrP^C^, Aβ30 and Cu^2+^ at equimolar ratios where A_∥_ = 171 and g_∥_ = 2.263. Consideration of the affinities from the glycine competition assay, Cu^2+^ should preferentially coordinate to PrP^C^ due to its higher affinity. However, while the combination spectrum preserves the superhyperfine splitting attributed to ^15^N-PrP^C^, the intensity is reduced. Moreover, there are subtle shifts in A_∥_ and g_∥_ with values distinct from either Cu^2+^-PrP^C^ or Cu^2+^-Aβ, which suggests that Cu^2+^ is in a new coordination environment with Cu^2+^ simultaneously coordinating with both Aβ30 and PrP^C^. To elucidate this new coordination environment and identify the coordinating atoms, we applied ESEEM and HYSCORE.

### 3-Pulse ESEEM analysis of PrP^C^ and A**β**30

**Figure 2:**
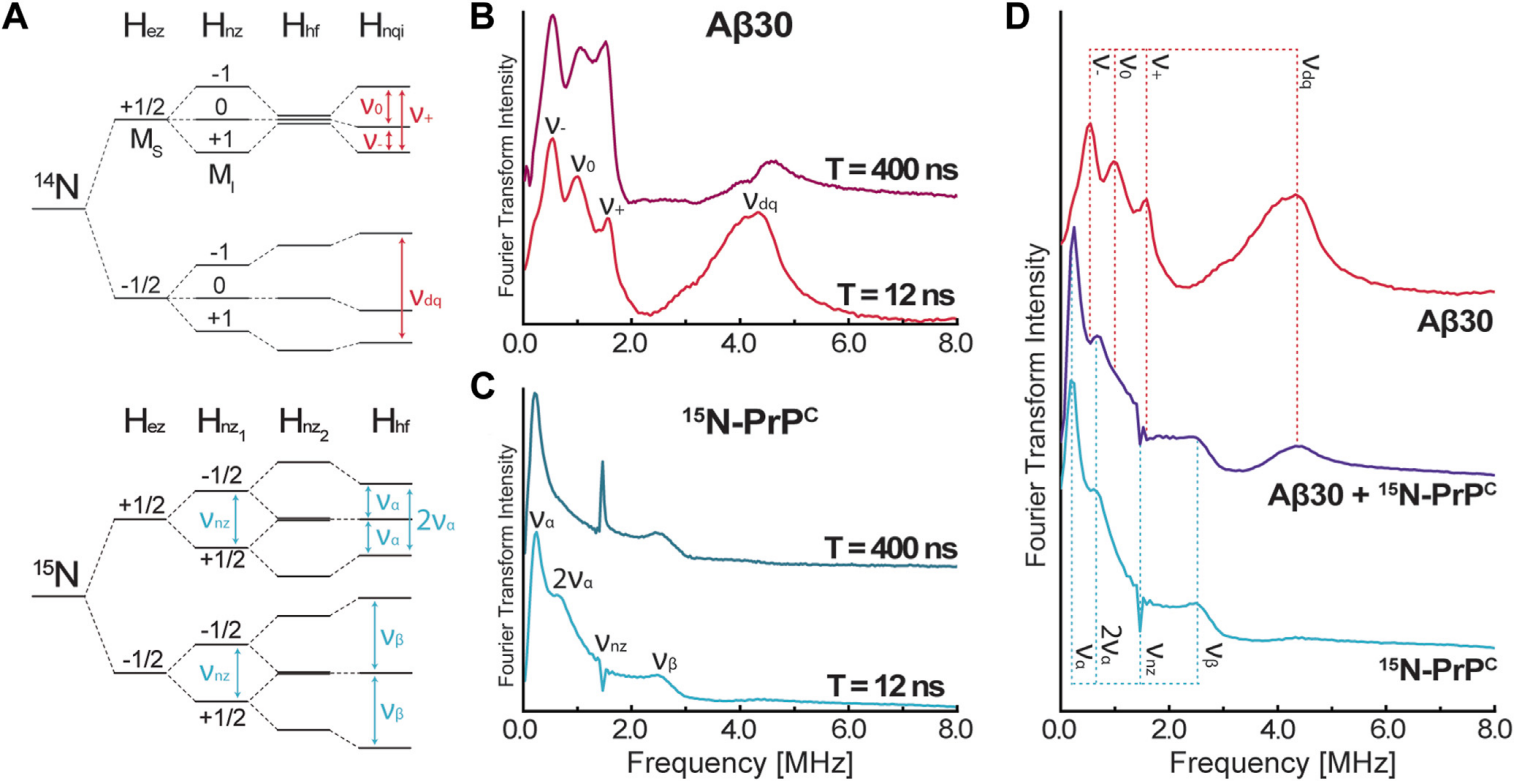
**3-Pulse ESEEM transitions observed for**^**14**^**N and**^**15**^**N His coordination.***A*, the energy level diagrams for the Cu^2+^ electron interacting with either one ^14^N or two ^15^N are displayed, along with their relevant ESEEM transitions. *B*, ESEEM frequency domain of Aβ30 with Cu^2+^ measured with two different initial T values, 400 ns or 12 ns. Both spectra show the three clear NQI peaks labeled as ν_−_, ν_0_, ν_+_ and DQ peak labeled as ν_dq_. *C*, ESEEM frequency domain of ^15^N- with Cu^2+^, performed under the same conditions. There are three identifying peaks labeled as ν_*α*_, ν_β_, and ν_nz_. A fourth peak is observed at approximately 2ν_*α*_. *D*, the T = 12 ns traces for natural abundance Aβ30 (*red, top*), ^15^N-PrP^C^ (*blue, bottom*), and ^15^N-PrP^C^ + Aβ30 (*purple, middle*) with equimolar amounts of Cu^2+^.

ESEEM provides important insight into the nuclei magnetically coupled to the Cu^2+^ center, revealing the nuclear Zeeman, hyperfine, and quadrupole interactions. One of the key advantages of ESEEM is its ability to differentiate between the nuclear spin environments of the isotopically labeled and unlabeled proteins. Since PrP^C^ is uniformly ^15^N-labeled (I = ½) its nuclear spin states will be distinct from those of the natural isotopic Aβ30 (I = 1), as shown in Figure 2*A*. Specifically, each coupled ^15^N exists in one of two nuclear spins states (M_I,_^15^N = ±½) whereas quadrupolar ^14^N exists among three states, (M_I,_^14^N = ±1, 0). At X-band frequencies, the nuclear Zeeman and hyperfine terms of ^14^N in the higher energy manifold are in exact cancelation, thus selectively revealing the nuclear quadrupole transitions (22, 44, 45). Conversely, the nuclear hyperfine term in the ^15^N energy manifold results in two observable transitions, ν_α_ and ν_β_. These transitions are described by Equations (1), (2), (3), (4), where A_iso_ is the isotropic hyperfine coupling, ν_nz_ is nuclear Zeeman frequency, T is the dipolar coupling, and θ is the angle between the electron-nuclear distance vector and the externally applied magnetic field (46, 47). By convention, we assign ν_α_ < ν_β_.

(1) $$\nu_{\alpha ,\beta }={\left[{\left(\nu_{nz}\pm \frac{A}{2}\right)}^{2}+{\left(\frac{B}{2}\right)}^{2}\right]}^{\frac{1}{2}}$$

(2) $$A=A_{iso}+T\left(3cos^{2}\theta -1\right)$$

(3) B=3Tsinθcosθ

(4) $$T=A_{iso}-A_{xx}=A_{iso}-A_{yy}=\frac{A_{zz}-A_{iso}}{2}$$

For histidine-Cu^2+^ coordination, relevant to our characterization here, ESEEM detects the noncoordinating nitrogen within the imidazole ring rather than the nitrogen directly coordinated to the paramagnetic center, outlined in Figure 1, *B* and *C* (48).

The ESEEM spectra for Aβ30 and ^15^N-PrP^C^ are shown in Figure 2, *B* and *C*, respectively. The spectrum of Aβ30 reveals two regions indicative of histidine-Cu^2+^ coupling (42, 49); the nuclear quadrupole interaction (NQI) peaks labeled as ν_−_ = 0.5 MHz, ν_0_ = 1.0 MHz, and ν_+_ = 1.5 MHz, along with the double quantum (DQ) peak, ν_dq_ = 4.2 MHz, consistent with previous work (21, 42, 49). Next, we observe four peaks in the ^15^N-PrP^C^ spectrum: ν_*α*_ = 0.3 MHz and ν_β_ = 2.5 MHz, consistent with previous work (50), ν_nz_ = 1.5 MHz, likely due to another weakly coupled ^15^N (*i.e.*, small A_iso_) and a new peak at 0.6 MHz that has not been previously identified in other literature. This peak is at 2× the frequency of the ν_*α*_ transition, which led us to investigate if this transition occurs when two or more equivalent ^15^N His are coupled to Cu^2+^. The ^15^N energy diagram (Fig. 2*A*) shows that when two equivalent ^15^N His are coupled to the Cu^2+^ center, there is a potential 2ν_*α*_ transition. Using the program EasySpin (51), we simulated 3-pulsed ESEEM spectra with varying numbers of equivalent ^15^N His coupled to Cu^2+^, as shown in Fig. S4. This analysis supports a 2ν_*α*_ transition occurring upon multiple equivalent ^15^N His coupling to Cu^2+^. Other possible sources for the 0.6 MHz transition, such as blind spots due to τ suppression and multiple nonequivalent ^15^N are explored in the subsequent section.

Before acquiring three-pulse ESEEM spectra of both ^15^N and ^14^N simultaneously, we set out to optimize pulse sequence parameters to provide the greatest intensity of all relevant frequency transitions. First, we examined how initial T changes the observed ESEEM spectra of Aβ30 and ^15^N-PrP^C^, as shown in Figure 2, *B* and *C* respectively. In other published studies, a longer initial T values are used to allow adequate spacing between pulses (52). However, by decreasing the initial T from 400 ns to 12 ns, as expected, we find that broad components assigned to multiple His coordination, notably the diagnostic 4.2 MHz DQ peak for ^14^N His and the 0.6 MHz for ^15^N, are properly resolved. Additionally, the shorter initial T does not compromise the three ^14^N NQI peaks. Therefore, we set the initial T to 12 ns for subsequent ESEEM as it is optimal for both ^14^N and ^15^N.

To optimize ESEEM acquisition with respect to τ, we developed a mathematical expression to incorporate two equivalent I = ½ nuclei in ESEEM from fundamental ESEEM equations, Equations S2–S12. Using Equation S12, we calculated the intensity of ν_*α*_, ν_β_, and 2ν_*α*,_ as a function of τ, shown in Fig. S5. From Fig. S5, the optimal τ values for ν_*α*_ are between 140 to 250 ns. We therefore ran ESEEM experiments at τ = 144 ns and τ = 210 ns, noting that both have the added benefit of suppressing high-frequency proton ESEEM (27, 47). Fig. S6 shows the comparison of τ = 144 ns and τ = 210 ns for both Aβ30 and ^15^N-PrP^C^. Notably, setting τ = 144 ns distorts the ^14^N peaks for Aβ30 and decreases the ν_β_ peak intensity for ^15^N-PrP^C^, as predicted from Equation S12 and shown in Fig. S5. However, setting τ = 210 ns resolves all relevant transitions, including the diagnostic 2ν_α_. We therefore chose a τ = 210 ns for all subsequent ESEEM. The experimental ESEEM time domain signals are shown in Fig. S7. Lastly, in optimizing T and τ, we did not focus on the ν_nz_ transition as it is not indicative of His coordination. Further analysis of this peak is discussed in the HYSCORE section.

In Figure 2*D*, we show the optimized ESEEM spectra for Aβ30, ^15^N-PrP^C^, each with one equivalent of Cu^2+^. The associated echo detected field swept spectra are in Fig. S8. The ESEEM of Cu^2+^ with both Aβ30 and ^15^N-PrP^C^, prepared in a 1:1:1 mixture, shows clearly the simultaneous features from both proteins, consistent with Cu^2+^ simultaneously coordinating to Aβ30 and PrP^C^. All ESEEM of Figure 2*D* are normalized to the echo intensity at T = 12 ns, therefore permitting analysis of the relative peak intensities among samples. With this normalization to echo intensity and, hence, concentration, we observe that the ^14^N ν_dq_ peak intensity in the 1:1:1 sample is persistent but decreased by 70% in the presence of equimolar ^15^N-PrP^C^. Noting that the intensity of the ν_dq_ transition is reflective of the number of coordinated ^14^N His imidizoles (53), this suggests that Aβ His coordination to Cu^2+^ is reduced from two or three His (as reported for the Aβ-Cu^2+^ complex depending on pH and buffer conditions (19)), to one His upon the addition of ^15^N-PrP^C^. Furthermore, the 2ν_*α*_ peak, indicative of multiple ^15^N-PrP^C^ His coordination, remains pronounced in the 1:1:1 combination sample. As noted in the previous section, the dissociation constant of the PrP^C^-Cu^2+^ complex is approximately 30× lower than that of the Aβ-Cu^2+^ complex. As such, the ESEEM spectrum of the 1:1:1 sample should be dominated by the interaction between PrP^C^ and Cu^2+^. Observation of a persistent ^14^N signal in this mixture is therefore supportive of a ternary complex with both proteins simultaneously coordinating to the Cu^2+^ center. (Note: For reference, Fig. S9 compares ^14^N and ^15^N histidine ESEEM at equal concentrations). This tentative assignment is further tested with 2D HYSCORE experiments.

### ^15^N/^14^N HYSCORE

The ^14^N and ^15^N signals from the ESEEM transitions below 1.5 MHz exhibit significant overlap—we therefore performed HYSCORE EPR, which is sensitive to the same coupled nuclear transitions as ESEEM but introduces an additional π pulse and evolution period, thereby providing a two-dimensional spectrum with an increased peak-to-peak resolution, along with correlations between nuclear spin manifolds (22, 27, 28).

**Figure 3:**
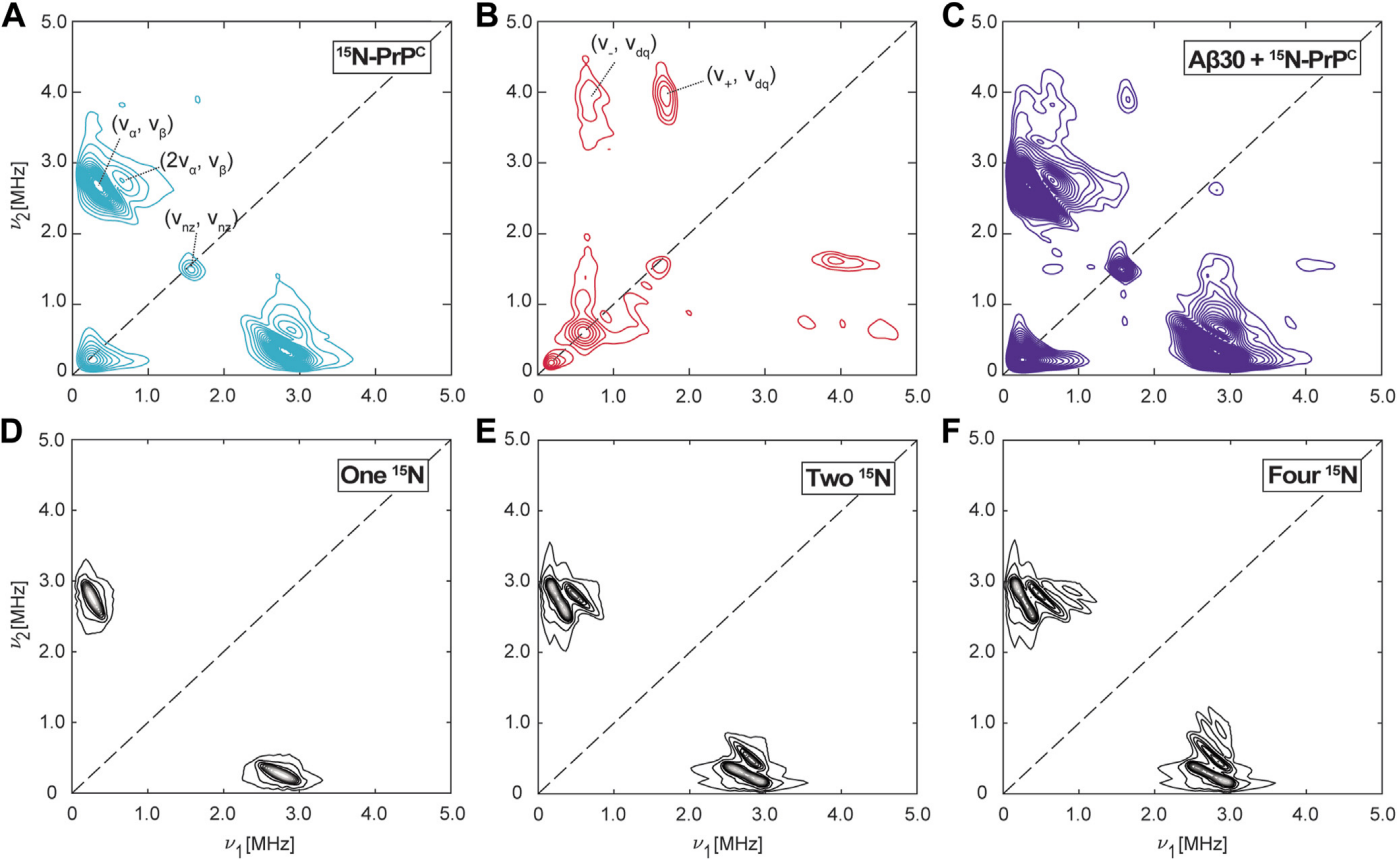
**HYSCORE Contour plots for**^**15**^**N and**^**14**^**N His coordination.***A*, ^15^N-PrP^C^ (*blue*), (*B*) natural abundance Aβ30 (*red*), and (*C*) Aβ30 + ^15^N-PrP^C^ (*purple*). Spectra were acquired with 100 μM Cu^2+^, 100 μM Aβ30, and 100 μM ^15^N-PrP^C^. The *black dashed line* represents the equation ν_1_ = ν_2_. *D*, simulated ^15^N HYSCORE spectra using one coupled ^15^N. τ was set to 210 ns to match the experimental τ. At this τ, only one peak is observed, implying no presence of a blind spot. Simulated HYSCORE with (*E*) two and (*F*) four coupled ^15^N nucleus were run with varied τ from 100 ns to 400 ns in 20 ns increments to account for any blind spots. Hyperfine values used to simulate these data are A_xx_ = A_yy_ = 3.2 MHz, A_zz_ = 2.0 MHz, A_iso_ = 2.8 MHz, and T = −0.4 MHz.

The HYSCORE spectrum of ^15^N-PrP^C^-Cu^2+^ is shown in Figure 3*A*, revealing intense cross peaks symmetric across the diagonal at (0.32 MHz, 2.68 MHz) and (2.68 MHz, 0.32 MHz), corresponding to (ν_α_, ν_β_), and the diagonally symmetric (ν_β_, ν_α_), respectively. Similar spectra have been observed for Cu^2+^ coordination to glycine (22) and histidine (49), as well as an interaction between organic radicals and histidine (54, 55). Proximal to these intense peaks, we also observe weaker transitions at (0.63 MHz, 2.74 MHz) and (2.74 MHz, 0.63 MHz) consistent with (2ν_α_, ν_β_) cross-peaks. Finally, a third peak is observed along the diagonal at (1.47 MHz, 1.47 MHz), which matches the ^15^N Larmor frequency. We associate the (1.47 MHz, 1.47 MHz) peak to weakly coupled ^15^N, as supported by Equations (1), (2), (3) in which ν_α_ and ν_β_ both converge to ν_nz_, in the limit of small A_iso_ and T. As shown in Fig. S10, HYSCORE simulations in this limit predict a weak ν_nz_ peak along the diagonal when compared to calculated off-diagonal peaks calculated for coupled ^15^N His. Furthermore, we observe that incorporating multiple weakly coupled ^15^N does not change the ν_nz_ peak, as seen in Fig. S11. Thus, this diagonal peak is assigned to the collective contribution from the rich bath of weakly coupled ^15^N existing at longer distances from the Cu^2+^ center throughout uniformly labeled PrP^C^.

Next, we obtained HYSCORE spectra from natural abundance Aβ30, with and without ^15^N-PrP^C^, as shown in Figure 3, *B* and *C*. These HYSCORE spectra were obtained with the same samples used in Figure 2. Focusing on natural abundance Aβ30, the reported HYSCORE contains cross peaks at (0.6 MHz, 3.8 MHz) and (1.6 MHz, 3.9 MHz), as well as similar peaks symmetric across the diagonal. These peaks are consistent with the (ν_−_, ν_dq_) and (ν_+_, ν_dq_) transitions observed when Cu^2+^ is coupled to the imidazole ring in histidine (22). Furthermore, the absence of a (ν_+_, ν_dq_) cross peak around (2.8 MHz, 4.0 MHz) indicates the absence of direct backbone amide coordination, consistent with current coordination models of Cu^2+^ to Aβ30 (10, 42). When both Aβ30 and ^15^N-PrP^C^ are present in the 1:1:1 mixture with copper (Fig. 3*C*), we are able to clearly resolve the aforementioned peaks (ν_−_, ν_dq_), (ν_+_, ν_dq_) from ^14^N-His, and (ν_α_, ν_β_) from ^15^N His. Importantly, the (2ν_α_, ν_β_) cross peak persists, demonstrating multiple ^15^N-His from PrP^C^ coordinated to Cu^2+^ in the presence of Aβ30. Together, Figure 3*C* reveals clear, distinguishable, simultaneous His-Cu^2+^ signals from both Aβ30 and ^15^N-PrP^C^, with the latter contributing at least two His to the complex.

Our interpretation of Cu^2+^ coordination to multiple His from PrP^C^ and at least one His from Aβ30 are reasoned on the grounds that the 2ν_α_ is due to Cu^2+^ coupling to two equivalent ^15^N. However, there are other possible sources for multiple peaks occurring in HYSCORE spectra. For example, the selection of a specific τ value can result in blind spots, in which certain cross peaks are split, giving the appearance of two adjacent peaks (27, 51, 56). To explore this possibility, we performed a HYSCORE simulation for a single coupled ^15^N incorporating the same τ used in the experiment. As shown in Figure 3*D*, the simulation results in a single peak with no observable blind spot. This is further examined in Fig. S12, which shows the results from several τ values. When ^15^N HYSCORE simulations do result in a blind spot, the observed cross peaks are not consistent with the (2ν_α_, ν_β_) cross peak observed experimentally.

Next, we performed HYSCORE simulations for two and four coupled ^15^N (Fig. 3, *E* and *F*), using the same parameters from Figure 3*D*. These simulations show that incorporation of equivalent ^15^N produces a progression of new, higher frequency peaks at multiples of ν_α_ and fixed ν_β_. Specifically, adjacent to the expected (ν_α_, ν_β_) is a cross peak at (2ν_α_, ν_β_), consistent with experimental spectra. This is in contrast to the observation of only a single cross peak reported in previous work done on systems with a single coupled ^15^N (22, 49, 55). Focusing next on the relative intensity of the observed cross peaks, the simulations find an intensity ratio (measured by peak height) of 1.0:0.6 between the original (ν_α_, ν_β_) and the (2ν_α_, ν_β_), which is higher than 1.0:0.3 observed experimentally. However, the ratios depend strongly on the broadening and the use of τ blind spot suppression in the simulations as well as the apodization used when processing the experimental HYSCORE data. Moving to four nuclei, the simulated HYSCORE contains a set of four peaks with an intensity ratio of 1.0:0.7:0.1:0.1. Because of the relatively low intensity of the latter two peaks, it would be difficult to observe these experimentally. Thus, it is feasible to differentiate between single and multiple ^15^N but determining the exact number of coupled ^15^N remains challenging through this analysis. Nevertheless, the assignment of at least two coupled ^15^N nuclei is clear in both the ^15^N-PrP-Cu^2+^ complex and the 1:1:1 Aβ, PrP^C^, Cu^2+^ mixture.

HYSCORE simulations in Fig. S13, show that the (2ν_α_, ν_β_) cross peak is resolved only when the magnitude of the dipolar coupling, T, is on the order of −0.4 MHz or greater. Simulations with a lower magnitude dipolar coupling, T = −0.1 MHz reveal solely the (ν_α_, ν_β_) cross peak. Moreover, at T = −1.2 MHz, a third cross peak appears at (2ν_α_, 2ν_β_), Fig. S14. Similar simulations with constant T but varying A_iso_ show little influence on the presence of (2ν_α_, ν_β_) or (2ν_α_, 2ν_β_) (Fig. S15). Based on the analyses above, we assign the (2ν_α_, ν_β_) peak observed in both ESEEM and HYSCORE to the ^15^N-His ΔI = ±2 transition, outlined in Figure 2*A*.

**Figure 4:**
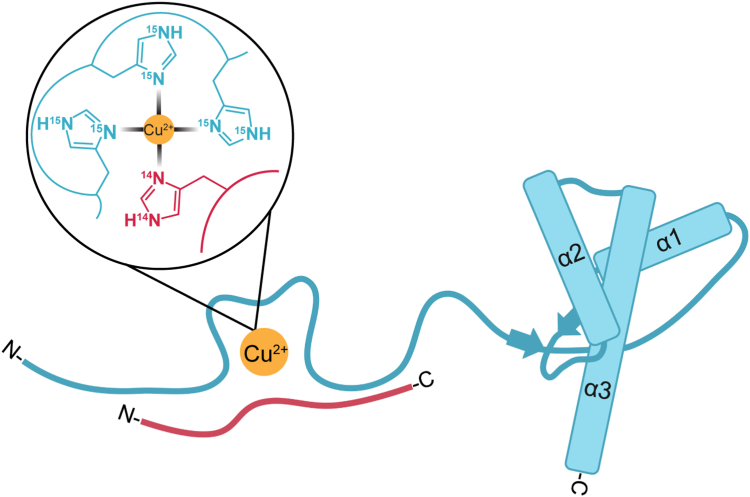
**Structural model of natural abundance Aβ and**^**15**^**N-PrP**^**C**^**coordinating to Cu**^**2+**^**.** The schematic structure of PrP^C^ is shown in blue, identifying its N-terminal IDP domain and its structured C-terminal domain, defined by three α-helices and one anti-parallel β-sheet. Our findings suggest that the disordered N-terminal domain coordinates Cu^2+^ through three ^15^N-His residues in the OR domain. Aβ is represented as an IDP (*red*) coordinating Cu^2+^ through a single ^14^N-His. The coordination of the three ^15^N-His and one ^14^N-His to Cu^2+^ is shown in the insert.

In this study, we employed various spectroscopic techniques, including CW-EPR, ESEEM, and HYSCORE, to examine and characterize Cu^2+^ coordination in the presence of both Aβ30 and PrP^C^ proteins. Our findings provide spectral evidence indicative of Cu^2+^ coordinating simultaneously with Aβ30 and PrP^C^. By CW-EPR, we identified that this coordination involves histidine residues from both proteins. By ESEEM and HYSCORE, we identified that when both proteins are present, there is a contribution from ^14^N and ^15^N histidine. Furthermore, we observed that upon the addition of PrP^C^ to Aβ30-Cu^2+^, the ^14^N-His ν_dq_ peak intensity decreases by 70%, suggesting a reduced His contribution from Aβ30. However, His coordination from Aβ persists despite its significantly lower affinity compared to that for the PrP^C^-Cu^2+^ complex. The (2ν_α_, ν_β_) assigned to two or more His from ^15^N-PrP^C^ remains in the presence of Aβ. Taken together, our data suggest that three His from PrP^C^ and a single His from Aβ30 coordinate to Cu^2+^ in the formation of a ternary complex. Our findings are summarized in Figure 4.

In addition to the characterization of this novel complex, we also developed a method that identifies multiple ^15^N-His coordination to copper centers and demonstrates the feasibility and utility of ^14^N and ^15^N mixed isotope experiments, especially as they apply to neurodegenerative proteins. This approach is readily adapted to segmentally labeled proteins, for example, to identify key residues or segments involved in metal regulation or chelation. Importantly, this study demonstrates that Aβ, PrP^C^, and Cu^2+^ interact and form a ternary complex at physiological pH, suggesting a potential mechanism for cellular uptake of Aβ, a crucial step in the progression of AD.

## Experimental procedures

### Protein expression and purification

The prion protein was expressed as previously described (32). In brief, the *M. musculus* PrP(23–230) construct cloned into the pJ414 vector (DNA 2.0) was transformed and expressed using *E. coli* (BL21 (DE3) Invitrogen). The protein was then purified following established methods (33). Bacteria were grown in M9 minimal media supplemented with ^15^N ammonium chloride (1 g/L) (Cambridge Isotopes) for uniformly ^15^N-labeled protein. Cells were grown at 37 °C until an OD_600_ of 1.0 was reached. Then, 1 mM isopropyl β-D-1-thiogalactopyranoside (IPTG) was added and cells were grown at 25 °C overnight. Proteins were extracted from inclusion bodies at room temperature with 8 M guanidinium chloride (GndHCl), 100 mM Tris, and 100 mM sodium acetate at pH 8. The soluble fraction was purified by Ni^2+^-immobilized metal-ion chromatography (IMAC). The column was washed and proteins were eluted with 5 M GndHCl, 100 mM Tris, and 100 mM sodium acetate at pH 4.5. The partially purified proteins were brought to pH 8 with 6 M potassium hydroxide (KOH) and left at 4 °C for 2 days to oxidize the native disulfide bridge. Finally, the proteins were desalted into 50 mM potassium acetate buffer and purified on the C8 column with reverse-phase liquid chromatography. The purified PrP^C^ was lyophilized and stored at −70 °C.

Aβ30 was transformed into a pET-28b(+) (Novagen) vector expressing His-tagged small ubiquitin-like modifier (His-SUMO) (Obtained from Professor Carrie Partch at UCSC) with Gibson cloning as previously described (57). Primers were purchased from Invitrogen to linearize His-SUMO DNA and create a linear fragment of Aβ30 DNA in pJ414 using Phusion High-Fidelity PCR Master Mix (New England Biolabs). Linearization reactions were run on a 1% agarose gel and extracted with GeneJET Extraction Kit (Thermo Fisher Scientific). Gibson reactions were run using Gibson Assembly Master Mix (New England Biolabs) and transformed into *E. coli* (DH5ɑ (DE3) Invitrogen). Colonies were grown and pure DNA was extracted using the Qiagen Mini prep kits and verified by DNA sequencing.

The construct was then transformed and expressed in *E. coli* (BL21 (DE3); Invitrogen). Cells were grown in Luria broth media (Research Product International) at 37 °C to an OD_600_ of 0.6 to 0.8. Then, 1 mM IPTG was added, and the cells continued to grow overnight at 18 °C. Next, the cells were harvested using a Sorvall Lynx 6000 centrifuge at 4 °C and 4000 rpm. Cells were resuspended in lysis buffer (50 mM Tris, 300 mM sodium chloride (NaCl), 1 mM β-mercaptoethanol (βME), and Pierce Protease Inhibitor Tablets (Thermo Fisher Scientific), pH 7.5). Afterwards, cells were sonicated using a FB505 Ultrasonic Processor with 15 s on, 30 s off pulses for 5 min. Cells were then centrifuged at 17,000 rpm for 45 min and purified using IMAC at 4 °C. The column was washed with 60 ml of Lysis buffer, followed by 30 ml of cleavage buffer (50 mM Tris, 50 mM NaCl, 30 mM Imidazole, 1 mM, pH 7.5). Proteins were incubated in column with 3 mM recombinantly expressed His-Ubl-specific protease one overnight at 4 °C. Protein was then eluted from the column with elution buffer (50 mM Tris, 50 mM Imidazole, βME, pH 7.5) and purified using HPLC on an Agilent PLRP-S column under basic conditions as previously described (4). Protein mass was confirmed using a SCIEX ExionLC liquid chromatography system in conjunction with a SCIEX X500B QTOF mass spectrometer, Fig. S1. Lastly, the purified Aβ30 was lyophilized and stored at −70 °C.

### Electron paramagnetic resonance

Lyophilized ^15^N-PrP^C^ was suspended in water, where Aβ30 was suspended in a small amount of 20 mM KOH and then diluted with water. The concentrations were calculated from the absorbance at 280 nm with an ε of 63,495.0 M^−1^·cm^−1^ and 1490.0 M^−1^·cm^−1^ for ^15^N-PrP^C^ and Aβ30 respectively. All samples were made to 100 μM for each protein, 100 μM Cu^2+^, 25 mM 4-(2-hydroxyethyl)-1-piperazineethanesulfonic acid (HEPES) buffer with 20% glycerol as a cryoprotectant (58), and the pH was adjusted to 7.4 with 100 mM KOH. After samples sat at room temperature for at least 15 min they were frozen in 4 mm quartz X-band tubes (Wilmad-LabGlass). For samples at pH 3.5, the 25 mM HEPES was substituted for 25 mM potassium acetate and the pH was adjusted with 100 mM HCl.

Continuous wave (CW)-EPR spectra were recorded at 121 K with a Bruker E500 CW-EPR spectrometer operating at X-band frequency (∼9.3 GHz) using an ER4122SHQE resonator (Bruker). CW-EPR data was simulated using EasySpin 5.1.10 toolbox within the MATLAB (The Mathworks Inc, Natick, MA) R2022b software suite (51).

Electron Spin Echo Envelope Modulations (ESEEM) and HYSCORE Spectroscopy time domain signals were acquired on an X-band (∼9.7 GHz) Bruker E580 EPR spectrometer using an MD4 resonator. Pulsed experiments were performed at the maximum of the Cu^2+^ spectrum, 3316 G. Furthermore, the experimental temperature was set to 18 K to optimize the signal-to-noise of Cu^2+^ (59). The 3-Pulsed ESEEM pulse sequence was π/2-τ-π/2-T-π/2-τ-echo (27) with four-step phase cycling. Here, we set τ to the hydrogen blind spot, which was calculated to be 210 ns for the magnetic field used (27, 47). The initial T values were set to 12 ns and incremented by 16 ns for 602 points and the π/2-pulse was 8 ns. Experiments using other T and τ values can be found in Fig. S7. After the acquisition, the ESEEM time domain was intensity normalized, fit to an exponential decay for background subtraction, zero-filled to 2048 points, and Fourier transformed using Bruker XEPR software. HYSCORE spectra were obtained using the pulse sequence π/2-τ-π/2-t_1_-π-t_2_-π/2-τ-echo (28). Here, τ was set to 210 ns to be consistent with the ESEEM experiments, π-pulse was 16 ns, and t_1_ and t_2_ were initially 40 ns with 32 ns increments for 256 points each. The HYSCORE spectra were collected using a four-step phase cycling. The resulting HYSCORE time domain signal was analyzed using Hyscorean (60). The time domain signals utilized Chebyshev apodization and were zero-filled to 1024 × 1024 points. Lastly, the HYSCORE spectra were obtained with a 2D Fourier transformed. Simulation of the HYSCORE spectra was obtained using the EasySpin toolbox in MATLAB R2022b (51, 61).

## Data availability

Raw data for EPR spectra in Figure 1, Figure 2, Figure 3 are available in https://github.com/Millhauser-Lab/Mixed-Isotope and also https://doi.org/10.5281/zenodo.14894932.

## Supporting information

This article contains supporting information.

## Conflict of interest

The authors declare no conflicts of interest.
