# Supplementary material for: Identifying the copper coordination environment between interacting neurodegenerative proteins: A new approach using pulsed EPR with 14N/15N isotopic labeling[image]
Source: J Biol Chem. 2025 Feb 13;301(3):108311. doi: 10.1016/j.jbc.2025.108311 (PMC11946511; doi:10.1016/j.jbc.2025.108311)
Supplement: Supporting information [file mmc1.docx]

Identifying the Copper Coordination Environment Between Interacting Neurodegenerative Proteins: A New Approach Using Pulsed EPR with ^14^N/^15^N Isotopic Labelling

Supporting Information

Amanda Smart, Kevin Singewald, Zikri Hasanbasri, R. David Britt, Glenn L. Millhauser

Table of Contents

Figure S1. SDS-PAGE and Mass Spectrometry confirmation of Aβ30 production …………… 3

Figure S2. Glycine competition assay to determine Cu^2+^ - protein binding affinities ………… 4

Figure S3. Comparison between ^14^N and ^15^N CW-EPR ……………………………………… 6

Figure S4. 3-pulse ESEEM spectra calculated from fundamental ESEEM equations and simulated in EasySpin …………………………………………………………………………………… 7

Figure S5. Simulation of ^15^N ESEEM frequency peaks intensities with respect to τ ………… 8

Figure S6. Comparison between τ values in ESEEM frequency domain of ^14^N and ^15^N ……… 9

Figure S7. Experimental ESEEM raw time domain signal …………………………………… 10

Figure S8. Echo detected field swept spectra …...…………………………………………… 11

Figure S9. ESEEM signal intensity comparison of ^14^N and ^15^N ……………………………… 12

Figure S10. Intensity comparison between strongly and weakly coupled ^15^N ………………… 13

Figure S11. Effect of multiple weakly coupled ^15^N nuclei in HYSCORE …………………… 14

Figure S12. Blindspot in HYSCORE simulations …………………………………………… 15

Figure S13. HYSCORE simulation with varying dipolar coupling ………………………… 16

Figure S14. HYSCORE simulation with large dipolar coupling …………………………… 17

Figure S15. HYSCORE simulation with varying hyperfine coupling ……………………… 18

Equation S1. Calculation for copper affinity (K_D_) in competition with bischelator ………… 4

Equation S2-S7. Time domain signal of a primary echo for two I = ½ nuclei ……………… 19

Equation S8. Full equation for a primary echo for two equivalent I = ½ nuclei …………… 20

Equation S9-S11. Time domain signal of a stimulated echo for two I = ½ nuclei …………… 20

Equation S12. Full equation for a stimulated echo for two I = ½ nuclei …………………… 21

**
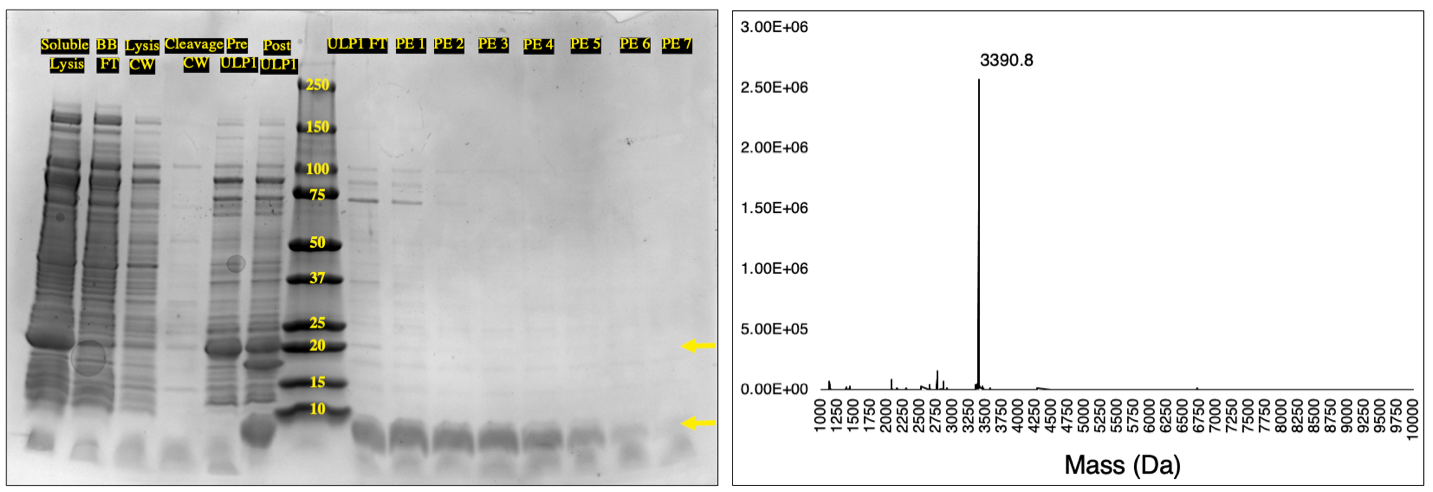
**

**Figure S1. SDS-PAGE and Mass Spectrometry confirmation of Aβ30 production.** SDS-PAGE (left) and mass spectrum (right) confirm the expression and purification of Aβ30. The gel shows lanes as stated from left to right: soluble cell lysate, Ni^2+^-IMAC batch binding flow through, lysis buffer column wash, cleavage buffer column wash, pre-addition of ULP1 SUMO-protease, post-addition of ULP1 SUMO-protease, ULP1-cleave reaction flow through, protein elution buffer fraction 1-7. The cleavage of SUMO-Aβ30 is seen by the appearance of a band at 17.1 kDa for SUMO and 3.4 kDa for Aβ30 in the Post-ULP1 lane. The SDS-PAGE gel was stained with SimplyBlue SafeStain and was captured on a Bio-Rad ChemiDoc imaging system, with the raw annotated image shown. The mass spectrum of purified Aβ30 shows that correct mass of 3390.8 Da.


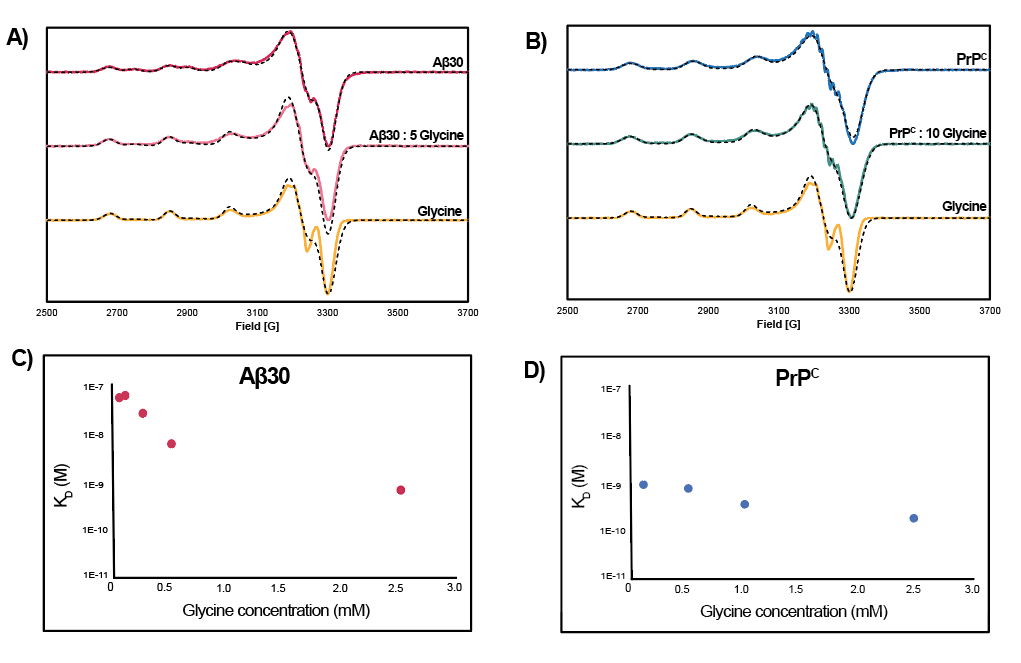


**Figure S2: Glycine competition assay to determine Cu^2+^- protein binding affinities.** CW-EPR was used to calculate K_D_ for Aβ30-Cu^2+^ and PrP^C^-Cu^2+^ in 50 mM HEPES, pH 7.4. Sample spectra for (A) Aβ30 and (B) PrP^C^ in competition with glycine: (top) spectrum of protein-Cu^2+^ complex, (middle) is protein: X glycine: Cu^2+^ complex, and (bottom) is glycine-Cu^2+^ complex. Each were recorded with 100 µM protein, 100 µM Cu^2+^, and 2500 µM glycine, except for in the protein glycine sample where glycine was 500 µM or 1000 µM, for Aβ30 or PrP^C^, respectively. The dashed spectra overlaid are the EasySpin simulated spectra. From the fitted simulations the percentage of protein bound to Cu^2+^ (PCu) could be determined and the respective K_D_ was calculated using the following equation, as previously described in (20):

$K_{Dc1}= \frac{\left[ C \right][Cu]}{[CCU]}$ ; $K_{Dc2}= \frac{\left[ C \right][Cu]}{[C_{2}CU]}$ ; $\beta=K_{Dc1}K_{Dc2}$; $K_{D}= \beta\frac{\left[ P \right][C_{2}Cu]}{[C]^{2}[PCu]}$ **(S1)**

It should be noted that glycine is a bischelator and binds in a 2:1 ratio with Cu^2+^. However, because the concentration of glycine is >> than K_Dc2_, the K_D_ of the protein can be related to the constant β, which is 1.7 × 10^−11^ M^2^ for glycine. The calculated K_D_ for each experimental condition is plotted for Aβ30 (C) and PrP^C^ (D). The calculated K_D_ for Aβ30 and PrP^C^ are 16.0 ± 14 nM and 0.519 ± 0.32 nM, respectively.


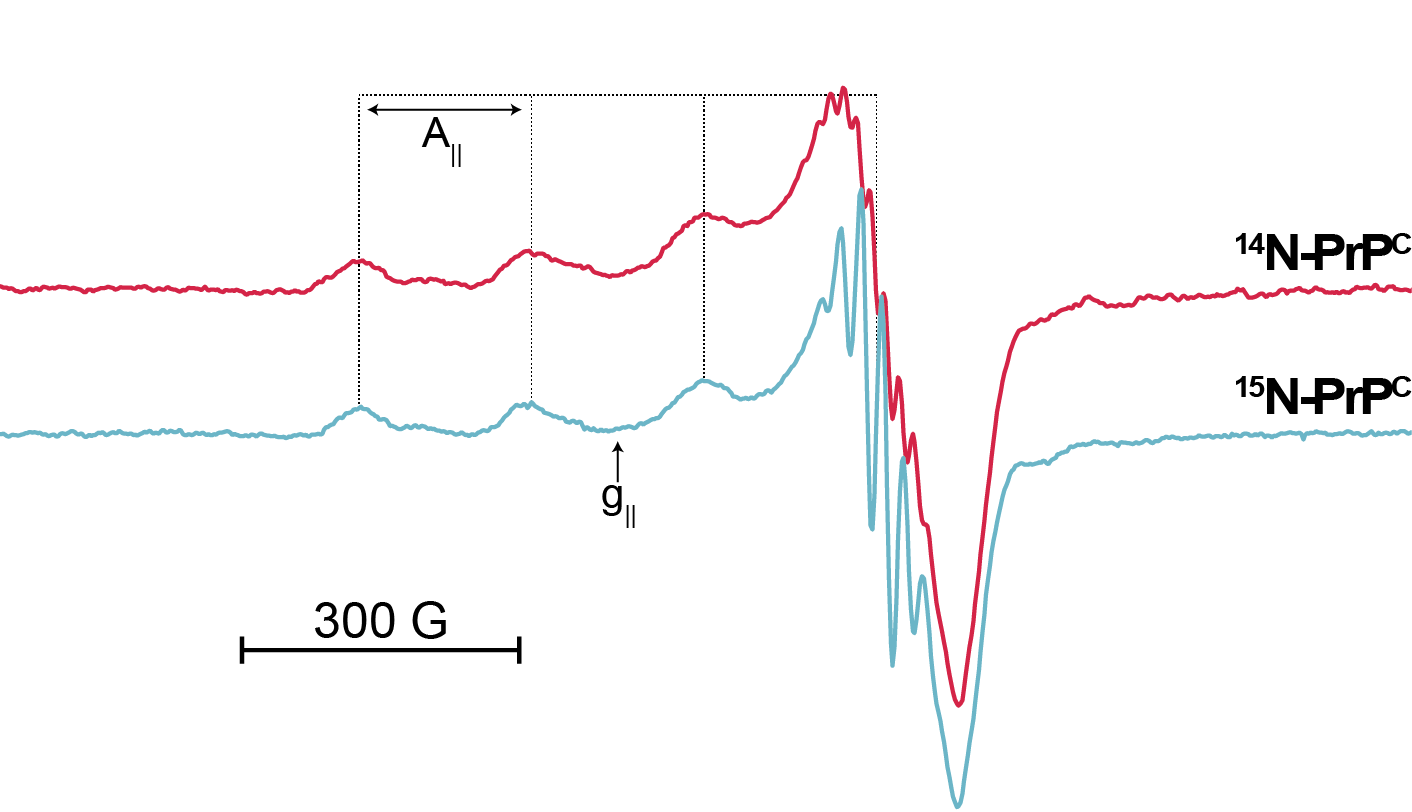


**Figure S3. Comparison between ^14^N and ^15^N CW-EPR.** Comparison between natural abundance ^14^N-PrP^C^ (red) and ^15^N-PrP^C^ (blue) CW-EPR. Both spectra have similar A_∥_ and g_∥_ values. One difference in the spectra is the superhyperfine observed in the high field perpendicular region. Because of the difference in nuclear spin, I_14N_ = 1 vs. I_15N_ = ½, the number of superhyperfine transitions changes. For four histidine coordination, ^14^N would give a much broader signal with 9 superhyperfine transitions, whereas ^15^N only has 5 transitions.


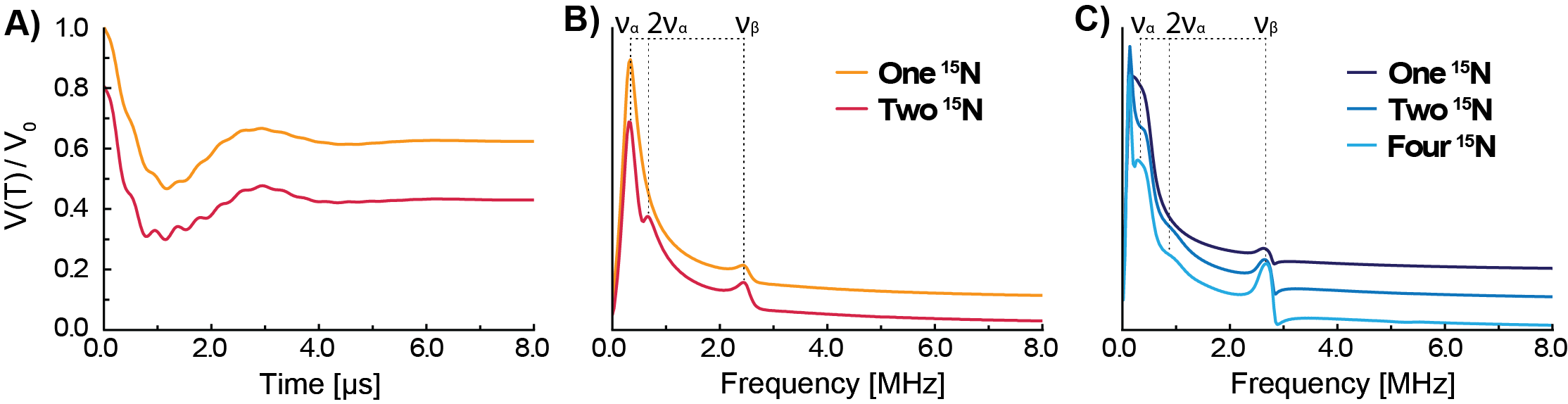


**Figure S4. 3-pulse ESEEM spectra calculated from fundamental ESEEM equations and simulated in EasySpin.** A) Calculated 3-pulse ESEEM time domain signal and B) respective Fourier transform of one (orange), and two (red) ^15^N from Equation S9 and S11. $\nu_{\alpha}=0.3$ MHz, $\nu_{\beta}=2.5 MHz$, $k=0.2$, $\tau=0.210 \mu s$, and multiplying the time domain signal by $e^{-0.8T}$ to incorporate line broadening. The resulting spectra reports similar features to the spectra observed in Figures 2D, S6, and S7. C) EasySpin simulated ESEEM spectra of one, two, and four ^15^N. A_xx_ = A_yy_ = 2.9 MHz, A_zz_ = 1.7 MHz, A_iso_ = 2.5 MHz, T = -0.4 MHz. Beyond a single ^15^N, an additional peak appears around twice that of ν_α_.

**
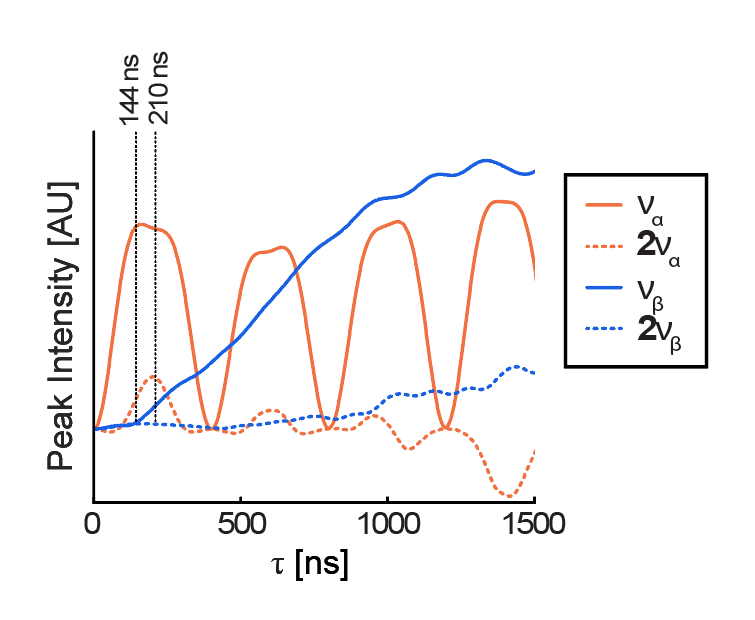
**

**Figure S5. Simulation of ^15^N ESEEM frequency peaks intensities with respect to τ.** Relative peak intensities for the ν_α_, ν_β_, 2ν_α_, and 2ν_β_ transitions calculated from Equation S12 at varying τ. The vertical lines represent the τ explored experimentally. Here, τ = 144 ns gives a high intensity ν_α_ but ν_β_ is negligible. This agrees with experimental results outlined in Figure S6. At τ = 210 ns, we anticipate a similar intensity of ν_α_, with a small ν_β_ and 2ν_α_ peak. 2ν_β_ cannot be observed until much longer τ. However, T_m_ relaxation would significantly influence the intensity of the ESEEM echo at these values.

**
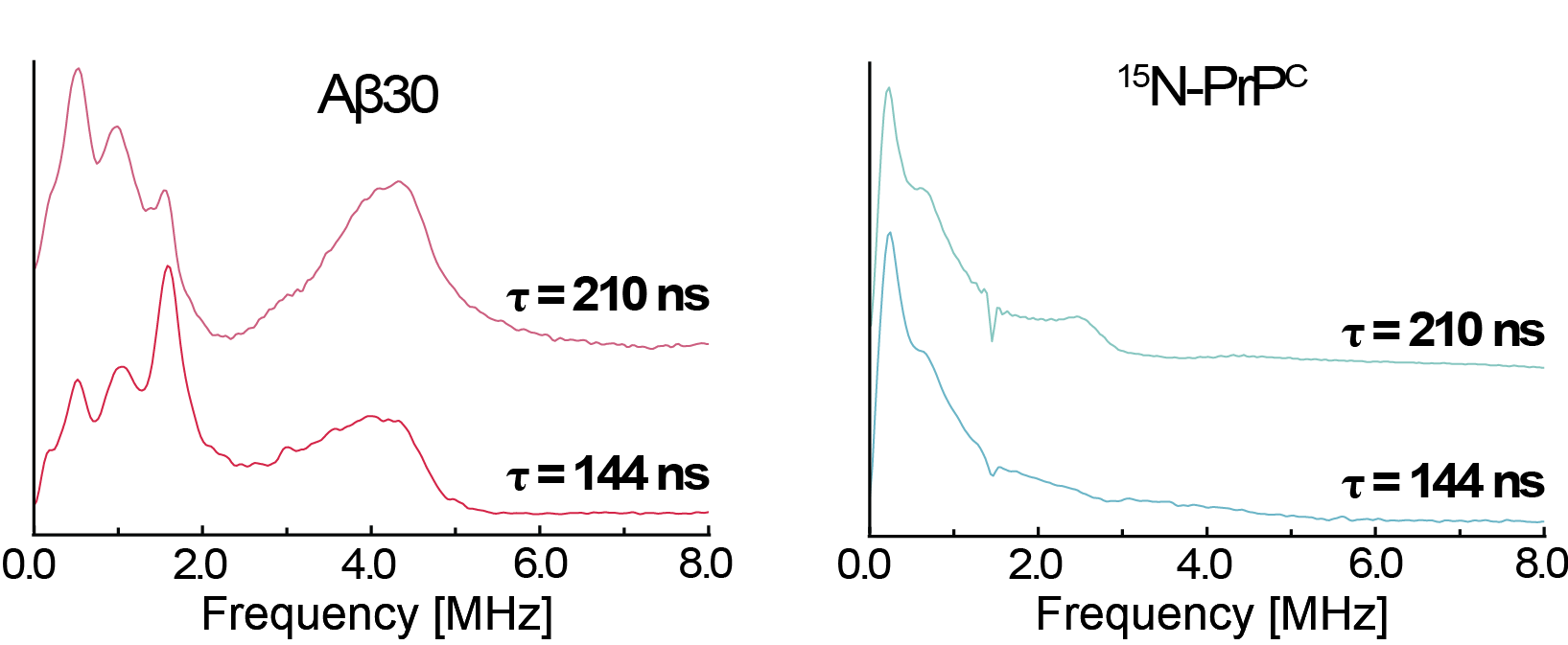
**

**Figure S6. Comparison between τ values in ESEEM frequency domain of ^14^N and ^15^N.** ESEEM frequency domain of Aβ30 (left) and ^15^N-PrP^C^ (right) show the comparison of samples obtained at a τ of 210 ns (top) and 144 ns (bottom), T initially equaling 12 ns. These τ were chosen to suppress the ^1^H signal at 3316 G. τ = 144 ns slightly distorts the ^14^N peaks and does not resolve the ν_β_ for ^15^N, as predicted by Figure S5. Thus, τ = 210 ns was used for subsequent experiments.

**
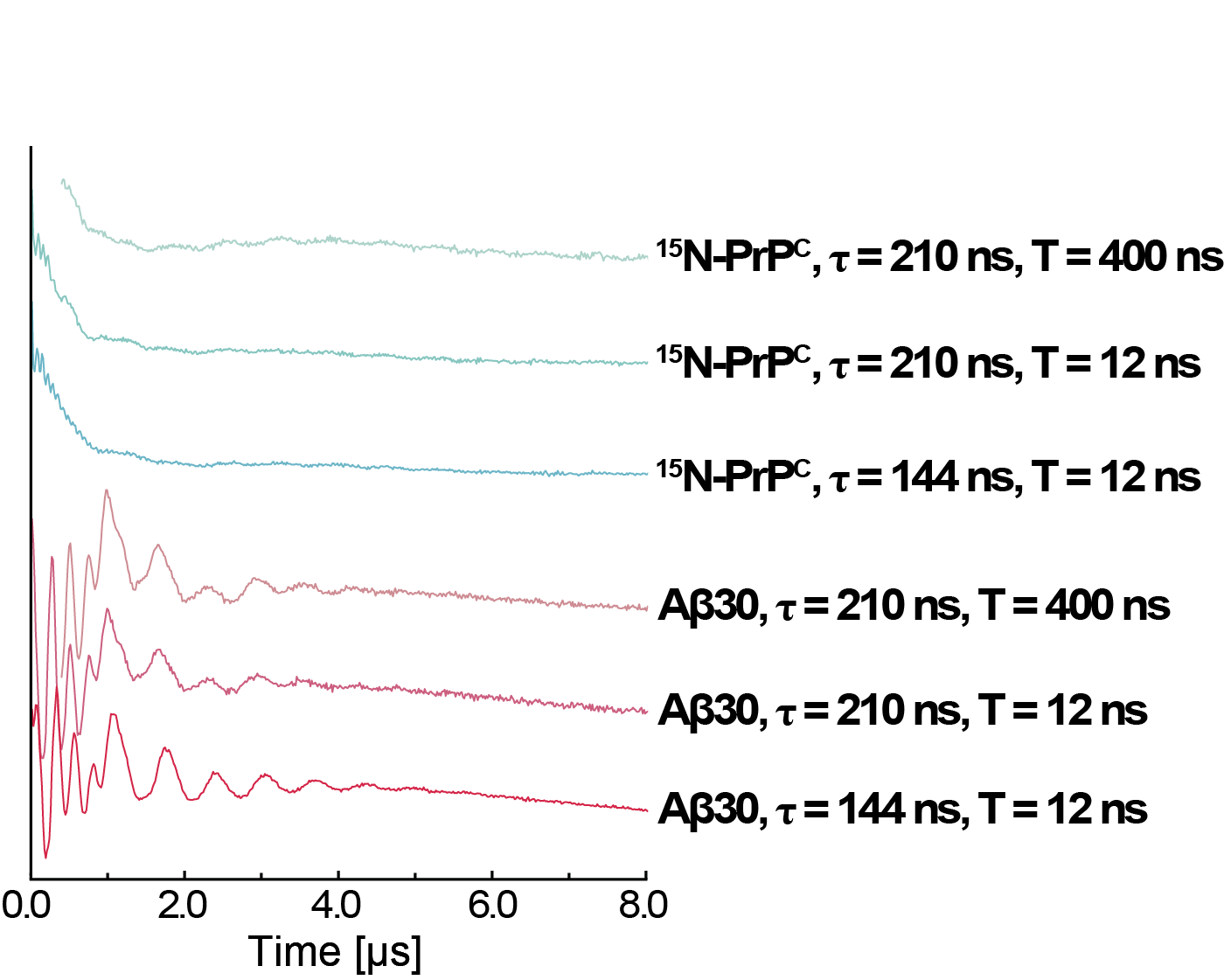
**

**Figure S7. Experimental ESEEM raw time domain signal.** ESEEM time domain signal of ^15^N-PrP^C^ (blue) and Aβ30 (red) at different τ and initial T values. T and τ values are reported with their respective time domain signals. The shortest T was 12 ns as that is the lower limit without significant distortion due to pulse overlap.

**
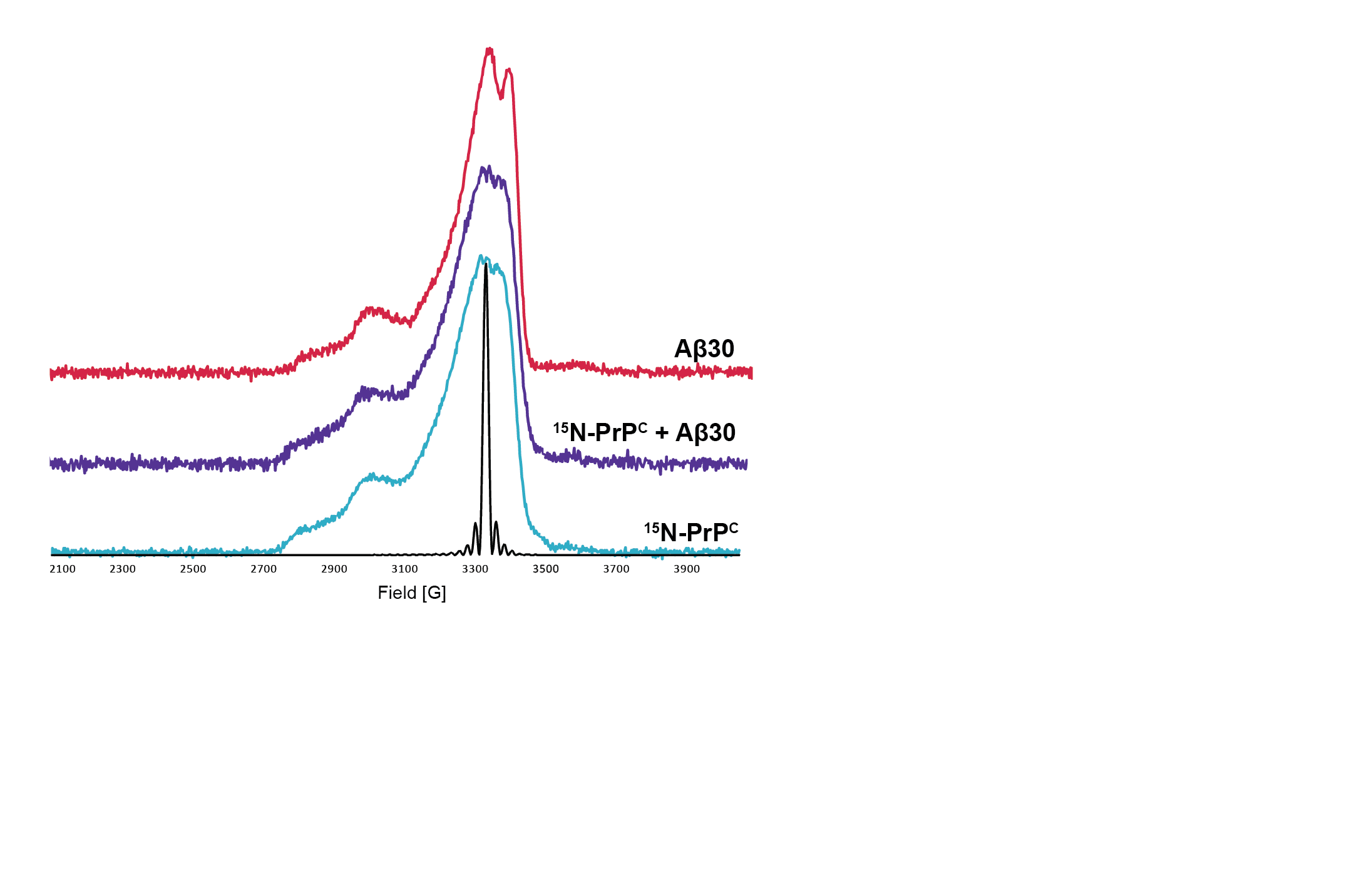
**

**Figure S8. Echo detected field swept spectra with the excitation profile of a 16 ns pulse.** The echo detected field sweep for Aβ30 (red), ^15^N-PrP^C^ + Aβ30 (purple) and ^15^N-PrP^C^ (blue) with 1 eq. Cu^2+^ are shown. The bottom black spectrum represents the excitation profile at 3361 G for a 16 ns π-pulse, which is used for HYSCORE experiments.

**
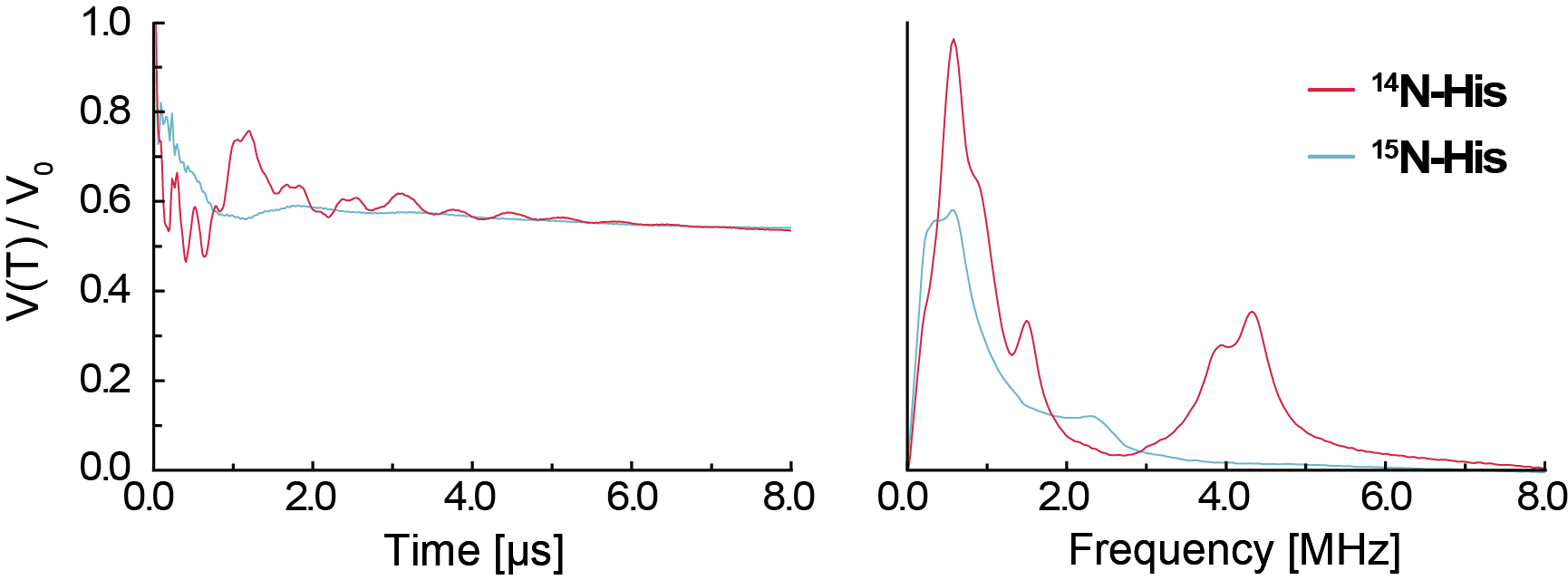
**

**Figure S9. ESEEM signal intensity comparison of ^14^N and ^15^N.** ESEEM time domain (left) and frequency domain (right) of ^14^N and ^15^N histidine in complex with Cu^2+^. The samples were prepared with a 1:4 ratio of Cu^2+^ : ^14^N or ^15^N histidine, with a Cu^2+^ concentration of 300 μM. The time domain signals were normalized to V(0) = 1 to account for subtle variations in sample prep, spectrometer tuning, and spectral changes between the two systems. The ^14^N nuclei yield a greater peak intensity compared to the ^15^N nuclei

**
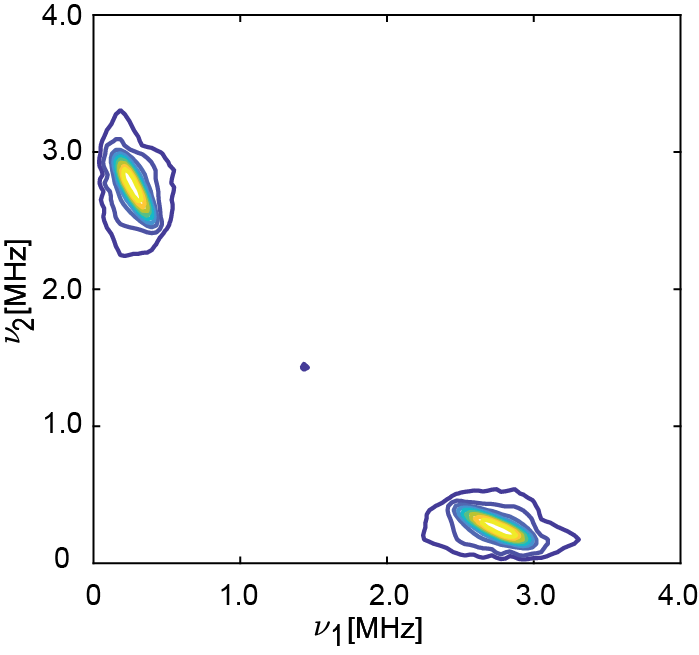
**

**Figure S10. Intensity comparison between strongly and weakly coupled ^15^N.** Simulated HYSCORE spectrum of Cu^2+^ interacting with one coupled and five weakly coupled ^15^N. Despite there being 5x weakly coupled ^15^N, the coupled ^15^N signal intensity if much larger. The strongly coupled ^15^N has an A_xx_ = A_yy_ = 3.2 MHz, A_zz_ = 2.0 MHz, A_iso_ = 2.8 MHz, T = -0.4 MHz and weakly coupled A_xx_ = A_yy_ = 0.1 MHz, A_zz_ = 0.01 MHz, A_iso_ = 0.07 MHz, T = -0.03 MHz. The small amount of anisotropy added to the weakly coupled hyperfine tensor was implemented to maximize its peak intensity without causing any observable splitting. Notably, the experimentally observed peak is significant. This is likely a consequence of one of two possibilities.

1. The peak might be due to coupling with a specific ^15^N within PrP^C^, giving rise to some nonnegligible, yet unquantifiable anisotropy, thus producing a significant signal.
2. There is an interaction with the bath of ^15^N, therefore stemming from the contribution from many weakly coupled ^15^N nuclei.

Since the number of nuclei does not influence peak position or shape for weakly coupled ^15^N, as shown in Figure S11, both are logical conclusions.

**
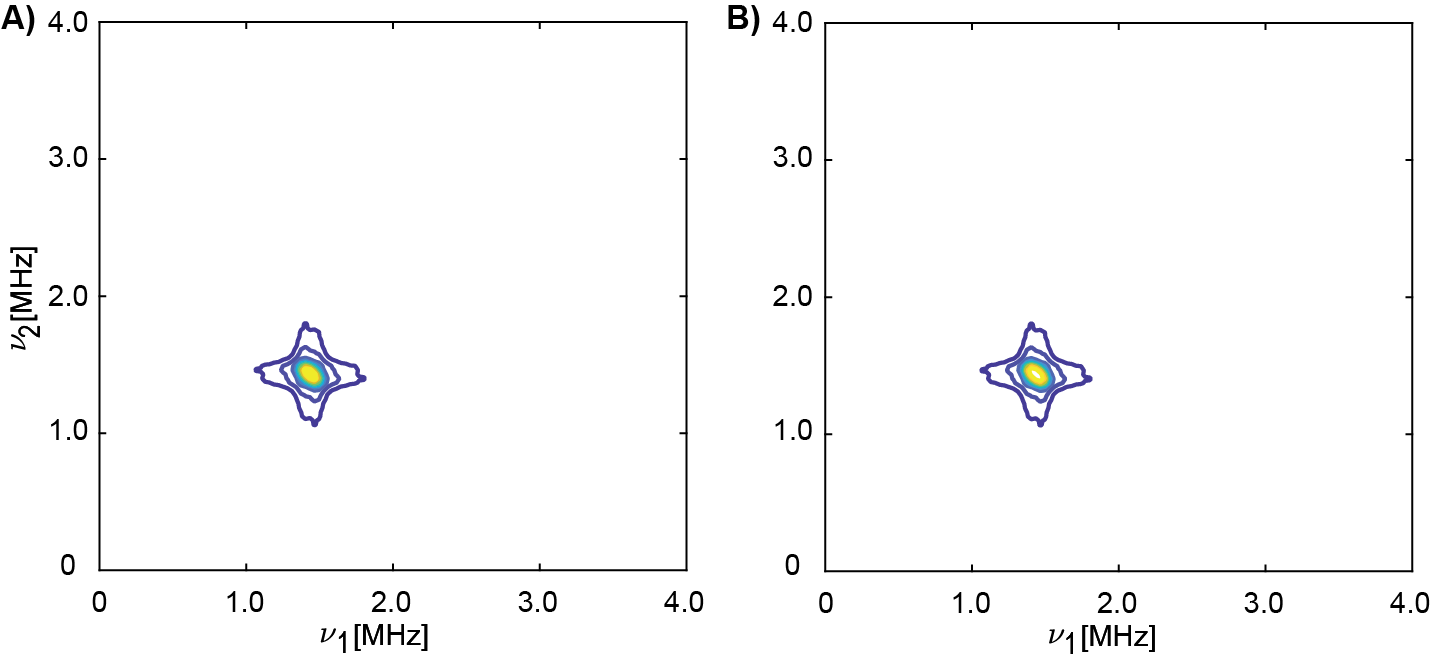
**

**Figure S11. Effect of multiple weakly coupled ^15^N nuclei in HYSCORE.** Simulated HYSCORE spectra of A) one and B) six weakly coupled ^15^N. The number of weakly coupled ^15^N has no observable effect on the simulated spectra. A_xx_ = A_yy_ = 0.1 MHz, A_zz_ = 0.01 MHz, A_iso_ = 0.07 MHz, T = -0.03 MHz. The small amount of anisotropy added to the weakly coupled hyperfine tensor was implemented to maximize the intensity of the weakly coupled peak without causing any observable splitting. Here, we observe a single peak along the diagonal at (1.45 MHz, 1.45 MHz). In comparison to the experimental HYSCORE, this peak lines up with the (1.47 MHz, 1.47 MHz) peak, confirming this peak is due to weakly coupled nuclei. Additionally, the presence of multiple weakly coupled nuclei does not influence peak position or shape. This is likely due to the low dipolar coupling, T, as shown in Figure S13.

**
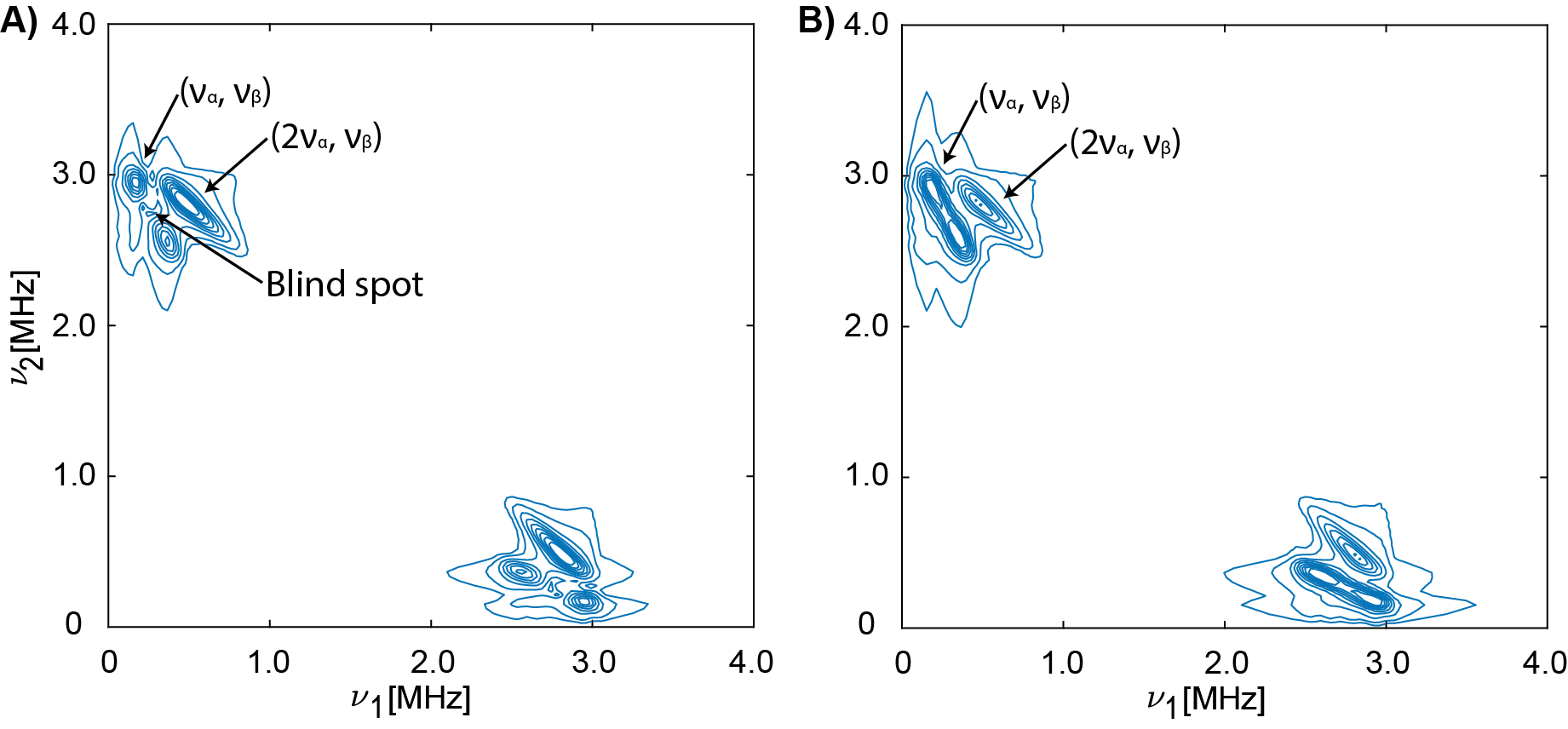
**

**Figure S12. Blindspot in HYSCORE simulations.** Simulated HYSCORE spectrum A) with τ = 210 ns and B) implementing τ averaging from τ = 100 to 400 ns in 10 ns steps. Without τ averaging, blind spots are possible. However, the frequencies of either of the two (2ν_α_, ν_β_) peaks resulting from the blind spot split do not match that observed for the (2ν_α_, ν_β_) peak. With two ^15^N and utilizing τ averaging, the resulting HYSCORE simulation best represents the experimental ^15^N-PrP^C^ in Figure 3.

**
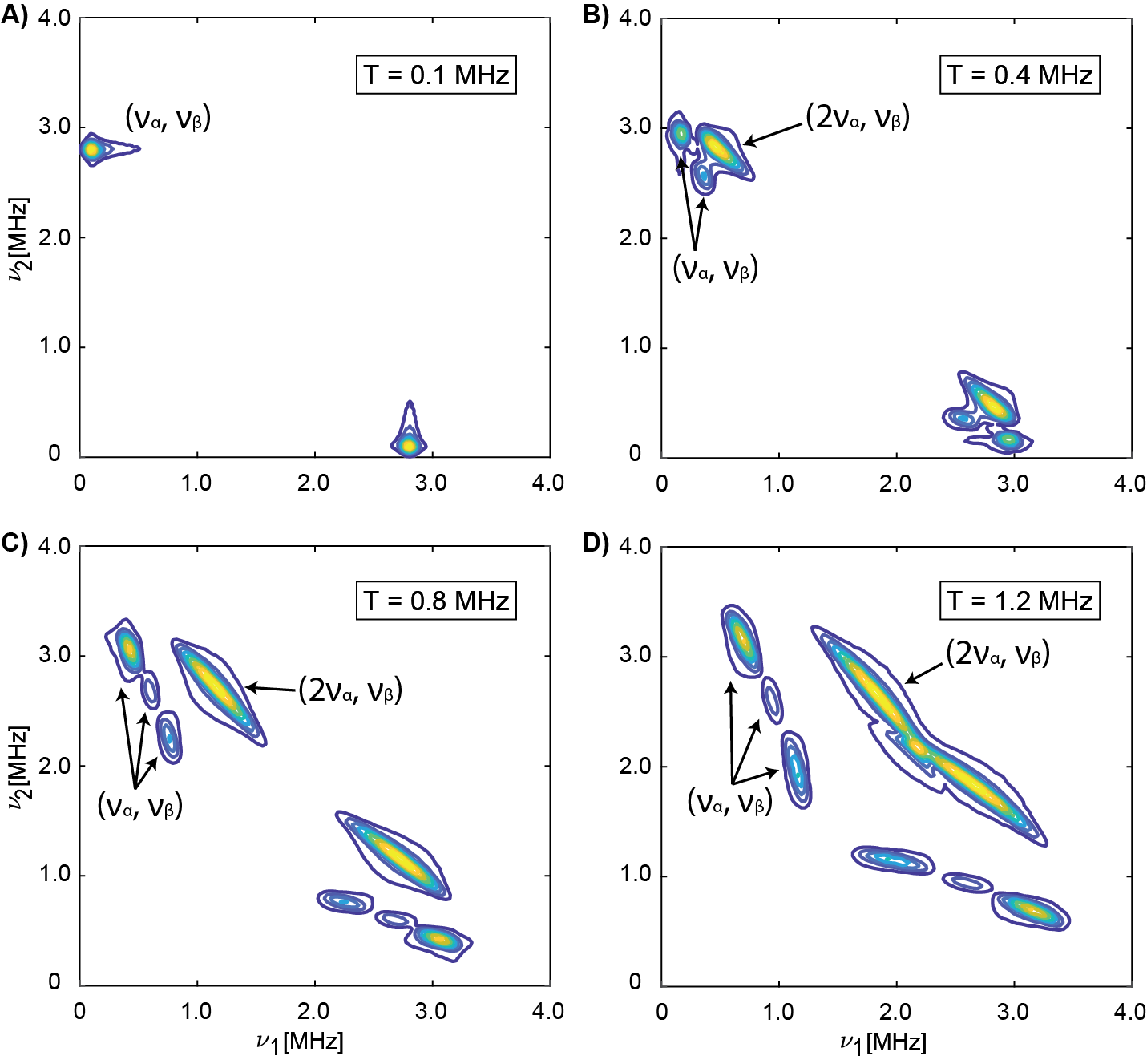
**

**Figure S13. HYSCORE simulation with varying dipolar coupling.** Simulated HYSCORE of two ^15^N nuclei with A_iso_ = 2.8 MHz, τ = 210 ns, A) T = -0.1 MHz, B) T = -0.4 MHz, C) T = -0.8 MHz, and D) T = -1.2 MHz. The gaps in the (ν_α_, ν_β_) cross peak are due to τ blind spots. For T = -0.1 MHz, only the (ν_α_, ν_β_) cross peak appears.

**
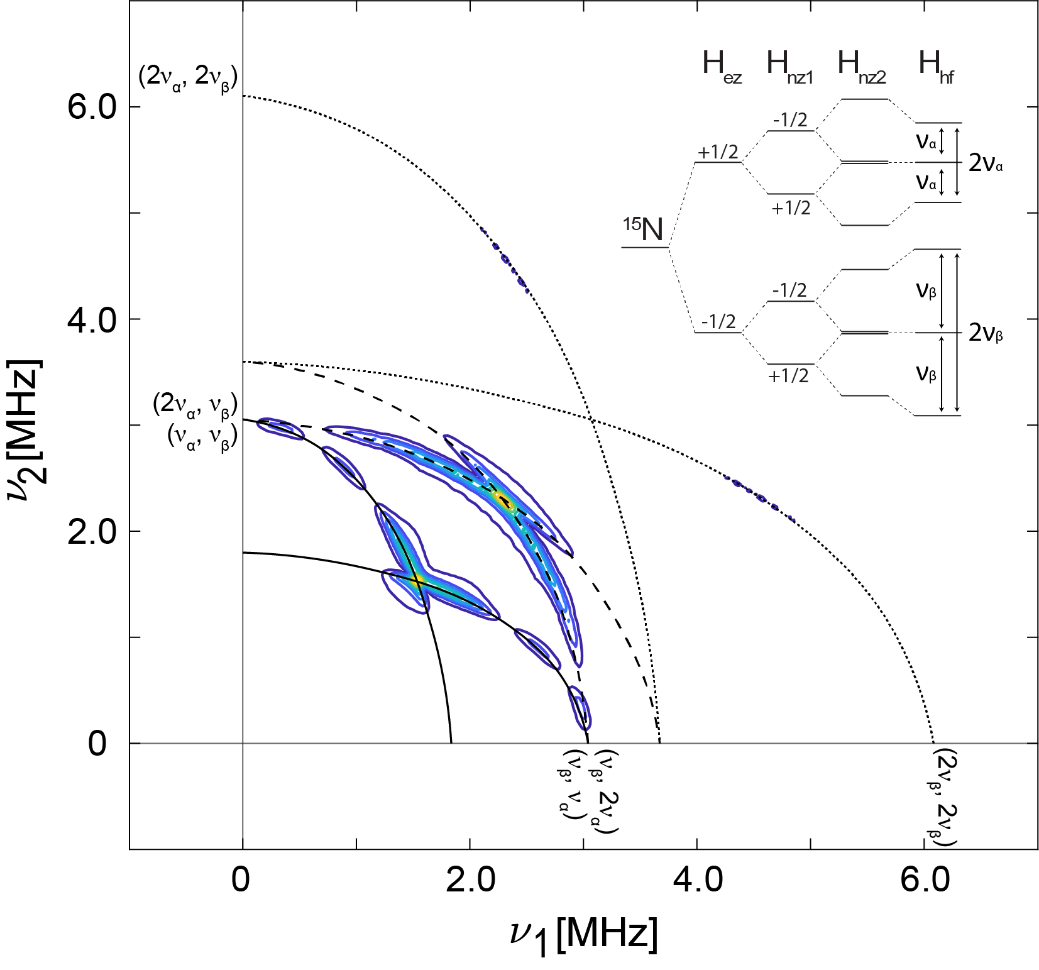
**

**Figure S14. HYSCORE simulation with large dipolar coupling.** Simulated HYSCORE of two ^15^N nuclei with A_iso_ = 2.0 MHz, T = -1.2 MHz, τ = 210 ns. The black solid line was set to overlay on the (ν_α_, ν_β_) cross peak. The dashed line is set to exactly twice ν_1_ of the solid line, i.e., would expect to overlay with the (2ν_α_, ν_β_) cross peak. Similarly, the dotted line is at twice ν_1_ and twice ν_2_ of the solid line, thus should overlay with (2ν_α_, 2ν_β_). The diagonally symmetric peaks are also fit in a similar manner. The inset contains the energy level diagram for a two ^15^N system. Vertical lines indicate the transitions observed in this HYSCORE simulation. By incorporating a much larger dipolar coupling in this simulation, in comparison to the T = -0.4 MHz used in Figure 3 and S11, the (2ν_α_, ν_β_) cross peak is more prominent and a new peak appears at (2ν_α_, 2ν_β_). Thus, it is likely these new peaks are dependent both on the presence of two ^15^N nuclei and a large dipolar coupling. The multiple peaks within the (ν_α_, ν_β_) cross peak are due to blind spots.


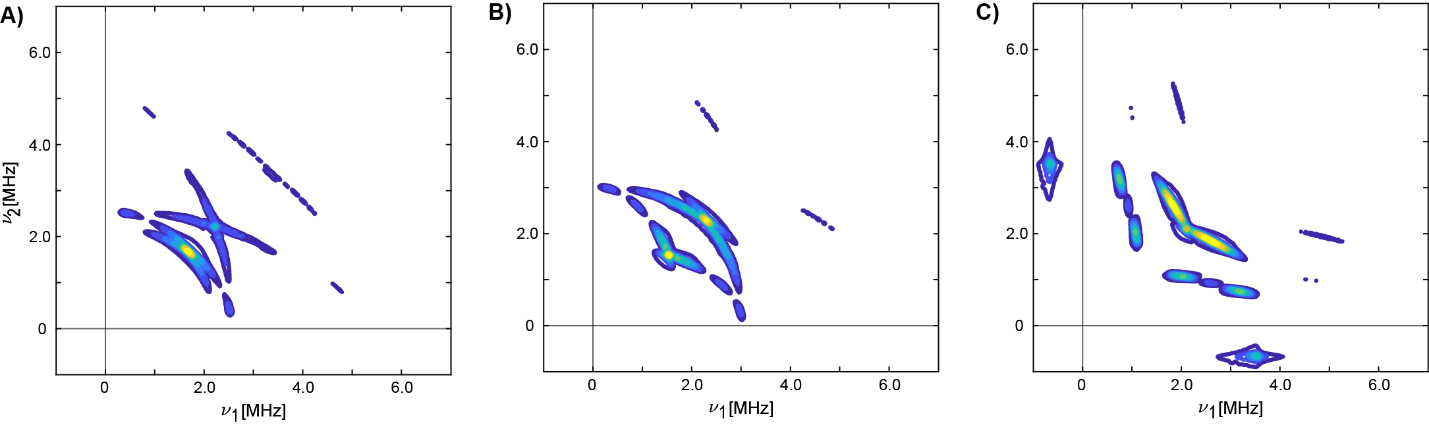


**Figure S15. HYSCORE simulation with varying hyperfine coupling.** Simulated HYSCORE spectra with an isotropic hyperfine coupling, A_iso_, of A) 1.0 MHz, B) 2.0 MHz, and C) 3.0 MHz. The dipolar coupling is held constant at T = -1.2 MHz. From this, we see that at high dipolar coupling, the cross peaks at (2ν_α_, ν_β_) and (2ν_α_, 2ν_β_) remain regardless of the A_iso_ used. Thus, these transitions are likely accessible primarily due to larger anisotropy, i.e., larger T, rather than a large A_iso_.

**Expressions for calculating ESEEM spectra.**

Equations S2 and S3 represent the time domain signal for a primary echo, V(τ) from 2-pulse ESEEM, for a single I = ½ nucleus. For two I = ½ nuclei, the resulting signal follows Equation S4, which can be simplified to the square of equation 8 when the nuclei are equivalent (27).

$V_{I=\frac{1}{2}}\left( \tau\right)=1-\frac{k}{2}A\left( \tau\right)$ **(S2)**

$A\left( \tau\right)=1-\cos\omega_{\alpha}\tau-\cos\omega_{\beta}\tau+\frac{1}{2}\cos{(\omega}_{\alpha}+\omega_{\beta})\tau+\frac{1}{2}\cos{(\omega}_{\alpha}-\omega_{\beta})\tau$ **(S3)**

$V_{I_{1}=\frac{1}{2}, I_{2}=\frac{1}{2}}\left( \tau\right)=V_{I=\frac{1}{2}}^{2}\left( \tau\right)=1-kA\left( \tau\right)+\frac{k^{2}}{4}A^{2}\left( \tau\right)$ **(S4)**

$k=\left( \frac{B\omega_{nz}}{\omega_{\alpha}\omega_{\beta}} \right)^{2}$ **(S5)**

$B=3Tcos\theta sin\theta$ **(S6)**

Here, k is the modulation depth parameter dependent on the dipolar coupling, ω is equal to 2πν, τ is the pulse timing, T is the dipolar coupling, and θ is the angle between the electron-nuclear vector and the direction of the applied magnetic field, B_0_. Upon a trigonometric expansion of Equation S4, a set of double frequency terms arise as shown in Equation S7. A full expansion is shown in Equation S8.

$V_{DF}\left( \tau\right)= k^{2}\left[ 3\cos2\omega_{\alpha}\tau+3\cos2\omega_{\beta}\tau+\frac{1}{2}\cos\left( {2\omega}_{\alpha}+{2\omega}_{\beta} \right)\tau+\frac{1}{2}\cos\left( {2\omega}_{\alpha}-{2\omega}_{\beta} \right)\tau\right]$ **(S7)**

It is evident from the modulation depth, ${3k^{2}}/{16}$, that the intensity of the double frequency transition is highly dependent on the dipolar coupling.

$V_{I_{1}=\frac{1}{2}, I_{2}=\frac{1}{2}}\left( \tau\right)= 1-k\left[ 1-\cos\omega_{\alpha}\tau-\cos\omega_{\beta}\tau+\frac{1}{2}\cos\left( \omega_{\alpha}+\omega_{\beta} \right)\tau+\frac{1}{2}\cos\left( \omega_{\alpha}-\omega_{\beta} \right)\tau\right]$ **(S8)**

$+ \frac{k^{2}}{4}\left[ 2.25-3\cos\omega_{\alpha}\tau-3\cos\omega_{\beta}\tau+2\cos\left( \omega_{\alpha}+\omega_{\beta} \right)\tau+2\cos\left( \omega_{\alpha}-\omega_{\beta} \right)\tau\right]$

$+ k^{2}\left[ 3\cos2\omega_{\alpha}\tau+3\cos2\omega_{\beta}\tau+\frac{1}{2}\cos\left( {2\omega}_{\alpha}+{2\omega}_{\beta} \right)\tau+\frac{1}{2}\cos\left( {2\omega}_{\alpha}-{2\omega}_{\beta} \right)\tau\right]$

$- \frac{k^{2}}{8}\left[ \cos\left( {2\omega}_{\alpha}+\omega_{\beta} \right)\tau+\cos\left( {2\omega}_{\alpha}-\omega_{\beta} \right)\tau+\cos\left( \omega_{\alpha}+{2\omega}_{\beta} \right)\tau+\cos\left( \omega_{\alpha}-{2\omega}_{\beta} \right)\tau\right]$

Equations S9 and S10 represent the time domain signal for a stimulated echo, V(τ,T) from a 3-pulse ESEEM, for a single I = ½ nucleus. For two, equivalent I = ½ nuclei, the resulting signal follows Equation S11.

$V_{I=\frac{1}{2}}\left( \tau,T \right)=\frac{1}{2}\left[ V_{\alpha}\left( \tau,T \right)+V_{\beta}\left( \tau,T \right) \right]$ **(S9)**

$V_{\alpha}\left( \tau,T \right)=1-\frac{k}{2}\left[ \left( 1-\cos\omega_{\beta}\tau\right)\left( 1-\cos\omega_{\alpha}\left( \tau+T \right) \right) \right]$ **(S10)**

$V_{\beta}\left( \tau,T \right)=1-\frac{k}{2}\left[ \left( 1-\cos\omega_{\alpha}\tau\right)\left( 1-\cos\omega_{\beta}\left( \tau+T \right) \right) \right]$ **(S10’)**

$V_{I_{1}=\frac{1}{2}, I_{2}=\frac{1}{2}}\left( \tau,T \right)=\frac{1}{2}\left[ V_{\alpha}^{2}\left( \tau,T \right)+V_{\beta}^{2}\left( \tau,T \right) \right]$ **(S11)**

Upon a trigonometric expansion of Equation S11, we obtain S12.

$V_{I_{1}=\frac{1}{2}, I_{2}=\frac{1}{2}}\left( \tau,T \right)=\frac{1}{2}[1-k\left( 1-\cos\omega_{\beta}\tau\right)+\frac{k^{2}}{4}\left( \frac{9}{4}-3\cos\omega_{\beta}\tau+\frac{3}{4}\cos2\omega_{\beta}\tau\right)$ **(S12)**

$+k\left( \left( 1-\cos\omega_{\beta}\tau\right)-\frac{k}{4}\left( 3-4\cos\omega_{\beta}\tau+\cos2\omega_{\beta}\tau\right) \right)\cos\omega_{\alpha}\left( \tau+T \right)$

$+\frac{k^{2}}{16}\left( 3-4\cos\omega_{\beta}\tau+\cos2\omega_{\beta}\tau\right)\cos2\omega_{\alpha}\left( \tau+T \right)]$

$+\frac{1}{2}[1-k\left( 1-\cos\omega_{\alpha}\tau\right)+\frac{k^{2}}{4}\left( \frac{9}{4}-3\cos\omega_{\alpha}\tau+\frac{3}{4}\cos2\omega_{\alpha}\tau\right)$

$+k\left( \left( 1-\cos\omega_{\alpha}\tau\right)-\frac{k}{4}\left( 3-4\cos\omega_{\alpha}\tau+\cos2\omega_{\alpha}\tau\right) \right)\cos\omega_{\beta}\left( \tau+T \right)$

$+\frac{k^{2}}{16}\left( 3-4\cos\omega_{\alpha}\tau+\cos2\omega_{\alpha}\tau\right)\cos2\omega_{\beta}\left( \tau+T \right)]$
